# Inhibition of Cathepsin B protects against vandetanib-induced hepato-cardiotoxicity by restoring lysosomal damage

**DOI:** 10.7150/ijbs.122904

**Published:** 2026-01-15

**Authors:** Wentong Wu, Jiangxia Du, Jinjin Li, Shaoyin Zhang, Xingchen Kang, Yashi Cao, Jian Chen, Zengyue Pan, Xiangliang Huang, Zhifei Xu, Bo Yang, Qiaojun He, Xiaochun Yang, Hao Yan, Peihua Luo

**Affiliations:** 1 Department of Cardiology, The Second Affiliated Hospital, Zhejiang University School of Medicine, Hangzhou, Zhejiang 310009, China.; 2 Innovation Institute for Artificial Intelligence in Medicine of Zhejiang University, Hangzhou 310018, China.; 3 Innovation in Digestive System Tumors, Ministry of Education, The Second Affiliated Hospital, Zhejiang University School of Medicine, Hangzhou 310017, China.; 4 Center for Drug Safety Evaluation and Research of Zhejiang University, College of Pharmaceutical Sciences, Zhejiang University, Hangzhou 310058, China.; 5 Institute of Pharmacology & Toxicology, College of Pharmaceutical Sciences, Zhejiang University, Hangzhou 310058, China.; 6 School of Medicine, Hangzhou City University, Hangzhou 310015, China.; 7 Nanhu Brain-computer Interface Institute, Hangzhou 311100, China.; 8 Hangzhou Institute of Innovative Medicine, College of Pharmaceutical Sciences, Zhejiang University, Hangzhou 310058, China.

**Keywords:** CTSB, lysosome dysfunction, autophagy inhibition, vandetanib, apoptosis

## Abstract

Vandetanib, a critical therapy for advanced thyroid and RET-driven cancers, is limited by life-threatening hepato-cardiotoxicity. This study identifies lysosomal protease cathepsin B (CTSB) as the central mediator of vandetanib-induced organ damage through STAT3-driven transcriptional activation. CTSB triggers mitochondrial apoptosis by cleaving the lysosomal calcium channel mucolipin TRP cation channel 1 (MCOLN1), disrupting calcium/AMP-activated protein kinase (AMPK) signaling and autophagy flux. Crucially, the natural compound tannic acid directly binds and inhibits CTSB, completely protecting against hepato-cardiotoxicity without compromising vandetanib's antitumor efficacy in preclinical models. Overall, our findings establish CTSB-mediated lysosomal dysfunction and MCOLN1-calcium-AMPK axis disruption as the core mechanism of vandetanib-induced hepato-cardiotoxicity, and identify tannic acid as a readily translatable adjuvant strategy to prevent this toxicity. These findings redefine CTSB as a druggable target for kinase inhibitor toxicities and position tannic acid as a clinically translatable adjuvant to enhance vandetanib's safety profile. By preserving lysosomal function and calcium homeostasis, this strategy addresses a critical unmet need in precision oncology, enabling prolonged, safer use of vandetanib and related tyrosine kinase inhibitors. The discovery of shared lysosomal injury mechanisms across organs also opens avenues for preventing multi-organ toxicities in broader cancer therapies.

## Introduction

Vandetanib is an oral tyrosine kinase inhibitor (TKI) targeting vascular endothelial growth factor receptor (VEGFR), epidermal growth factor receptor (EGFR) and rearranged during transfection (RET). It is indicated for the treatment of symptomatic or progressive medullary thyroid cancer in patients with unresectable locally advanced or metastatic disease 1. Additionally, it can effectively prolong the progression-free survival of RET fusion-positive advanced non-small cell lung cancer patients 2. However, severe hepato-cardiotoxicity significantly limits its clinical benefits. Vandetanib carries a black box warning for QT interval prolongation, Torsades de pointes, and sudden death. A fatal cardiac failure with pathological changes, including cardiomyocyte hypertrophy and marked myocyte degeneration in the subendocardial zones and papillary muscles of the myocardium, has been reported in patients taking vandetanib 3. A meta-analysis that included 45 randomized controlled trials including 20,027 patients showed that vandetanib had the highest risk of cardiotoxicity among all VEGFR TKis 4. Besides, elevated ALT levels have been detected in 50% patients, with 2% to 5% of these individuals exhibiting levels exceeding five times the normal threshold 5, 6. The mechanism of hepato-cardiotoxicity caused by vandetanib is still obscure with a lack of effective intervention strategies. It is a very clear unmet clinical need to be solved.

Lysosomes​​, now recognized as multifunctional signaling hubs beyond degradation, coordinate metabolism, autophagy, and immune surveillance 7. Dysfunctional lysosomal hydrolases drive pathological accumulations of amyloid-beta (Aβ), Tau, and alpha-synuclein (α-syn) in neurons, contributing to Alzheimer's and Parkinson's diseases; similarly, ​​premature zymogen activation by cathepsins​​ triggers pancreatic injury through vacuolar rupture in acinar cells 8. Beyond classical storage disorders, lysosomal impairment promotes ​​cancer progression and autoimmunity​​ via aberrant signaling or defective clearance 9, 10. Critically, lysosomal enzymes exhibit ​​context-dependent pleiotropy​​, mediating both cytoprotective and cytotoxic pathways through dynamic subcellular localization and stress-responsive mechanisms.

Cathepsins (CTS), a superfamily of lysosomal proteases comprising approximately 12 subfamilies, predominantly exhibit optimal enzymatic activity under the slightly acidic microenvironment of lysosomes. Among these, CTSB has emerged as a particularly well-characterized member, structurally consisting of a disulfide-linked heterodimer comprising heavy and light chains. Initial studies established its pathological roles in pancreatic injury and oncogenesis 11, 12, with subsequent mechanistic work revealing CTSB's critical mediation of cell death pathways across tissue injury models. Pathophysiologically, CTSB upregulation correlates strongly with: (1) hepatic fibrogenesis and cirrhosis progression via TGF-β activation 13, 14, and (2) cardiomyocyte necroptosis in ischemic heart disease through BAX-mediated mitochondrial dysfunction 15, 16. Paradoxically, in Alzheimer's disease, CTSB exhibits dual neurobiological effects—early-stage neuroprotection via Aβ plaque degradation 17, 18 versus late-stage disease exacerbation through IL-1β-dependent neuroinflammation 19, 20. This pleiotropy, driven by context-dependent subcellular localization and pathway selectivity, necessitates tissue-specific mechanistic investigations. Our study focuses on CTSB's role in liver/heart homeostasis and pathologies linked to its aberrant upregulation.

Given that mitochondrial apoptosis is a central execution pathway in diverse forms of tissue injury and drug-induced toxicity, we hypothesized that vandetanib-induced hepato-cardiotoxicity might also involve mitochondria-dependent apoptotic signaling 21, 22. In this study, we identify vandetanib-induced multiorgan toxicity as driven by STAT3 activation (Tyr705 phosphorylation), which upregulates CTSB to cleave the lysosomal calcium channel MCOLN1, disrupting calcium/AMPK signaling and triggering mitochondria-dependent apoptosis. CTSB-mediated MCOLN1 proteolysis emerged as the common trigger for both hepatotoxicity and cardiotoxicity, validated across dual-organ models. Finally, we demonstrate that inhibiting CTSB by tannic acid is a potential therapeutic strategy for vandetanib-induced hepato-cardiotoxicity.

## Materials and Methods

### Animal experiments

All procedures involving mice were in accordance with the guidelines set by the Institutional Animal Care and Use Committee of the Innovation Institute for Artificial Intelligence in Medicine at Zhejiang University. Male C57BL/6J mice, aged 6 to 8 weeks and weighing approximately 18 to 22 grams, were sourced from Beijing Vital River Laboratory. They were housed under controlled conditions with a 12-hour light/dark cycle, temperatures maintained between 21°C and 23°C, and humidity levels of 40% to 70%.

For constructing a mouse model of vandetanib-induced hepato-cardiotoxicity, C57BL/6J mice were randomly divided into control and dosing groups. The mice were given 0.5% carboxymethyl cellulose sodium (CMC-Na, Sinopharm, K2190201) or 100 mg/kg vandetanib (R11056, Shanghai Rechemscience Co., Ltd.) daily for 4 weeks. At the experimental endpoint, mice were echocardiographic tested, weighed, and collected blood samples, liver tissues, and heart tissues.

The *Atg7*^flox/flox^ mice were supplied by the RIKEN BioResource Research Center. The genetic background of these mice is as follows: B6. Cg-ATG7 <tm1Tchi>. Additionally, transgenic mice carrying the ALB-Cre gene, which expresses the Cre recombinase enzyme under the regulation of the albumin promoter, were sourced from the Shanghai Research Center for Model Organisms. *Atg7*^flox/flox^ mice were crossed with ALB-Cre mice to generate hepatocyte-specific *Atg7* knockdown mice. The progeny (*Atg7*^flox/+^; Alb-Cre) are hereafter designated *Atg7*^Δhep/+^ mice.

For verifying the effect of vandetanib-induced the up-regulation of CTSB, specific knockdown of CTSB in mouse hepatocytes is achieved by adeno-associated virus (AAV). A liver-specific adeno-associated virus serotype 9 (AAV9), allowing for hepatocyte-targeted and cardiac-targeted RNAi against Ctsb (AAV9-U6-sh*Ctsb*) was constructed and packaged by Shanghai Genechem Co., Ltd. The AAV9-control and AAV9-sh*Ctsb* were then injected into C57BL/6J mice through the tail vein.

For tannic acid intervening in vandetanib-induced liver and cardiac toxicity, male mice were administered 100 mg/kg vandetanib and/or 30 mg/kg tannic acid dissolved in 0.5% CMC-Na every day for 4 weeks. At the experimental endpoint, the mice were weighed, and the blood samples, liver samples, and heart samples were collected.

### Cell culture and treatment

HL-7702 and H9c2 cells were acquired from Guangzhou Jennio Biological Technology. The CCC-HEH-2 were provided by National Infrastructure of Cell Line Resource of China. The HEK-293T and AML12 cell lines were provided by Stem Cell Bank at the Chinese Academy of Sciences. Human primary hepatocytes were supplied by BioreclamationIVT, identified by the code XSM.F00995-P. Mouse primary hepatocytes (MPH) were extracted from male C57BL/6J mice aged between 6 to 8 weeks. All cell types were cultured under constant conditions at 37 °C with a 5% CO_2_ environment.

HL-7702 cells were cultured in RPMI-1640 medium (31800, Gibco) while the CCC-HEH-2, H9c2, HEK-293T, and AML12 cells along with MPH, were grown in DMEM medium (12800, Gibco) supplemented with 10% fetal bovine serum (16140071, Gibco), 100 U/mL penicillin (T5602, Topscience), and 100 µg/mL streptomycin (T75320, Topscience). For thawing AML12 cells, dexamethasone (ST1254, Beyotime) with a final concentration of 40 µg/L and ITS-X Media Supplement (100x) (C0345, Beyotime) were added to the medium. For thawing human primary hepatocytes, INVITROGRO^TM^ CP Medium (S03316, BioreclamationIVT) with 10% fetal bovine serum (16140071, Gibco) was used, followed by a 24-hour incubation. The medium was then replaced with INVITROGRO^TM^ HI Medium (Z99009, BioreclamationIVT) for continued hepatocyte culture.

The Research Resource Identifiers (RRIDs) for all cell lines used in this study are comprehensively documented in Supplementary Table 1.

Vandetanib (R11056) was provided by Shanghai Rechemscience Co., Ltd. Z-VAD-FMK (T7020), Ferrostatin-1 (Fer-1, T6500), Necrostatin-1 (Nec-1, T1847), Doxorubicin (T1456), Erastin (T1765), Bafilomycin A1 (BafA1, T6740), Chloroquine (CQ, T8689), NH_4_Cl (T64755), STAT3-IN-13 (T62491), Apigenin (T2175), Baicalein (T2858), and Tannic acid (T0801) were purchased from Topscience. CA-074 (CTSB inhibitor, HY-103350) was purchased from MedChemExpress. Necroptosis Inducer Kit with TSZ (C1058S) was purchased from Beyotime.

### RNA-seq transcriptome analysis

RNA was isolated from mice administered with CMC-Na or 100 mg/kg/day vandetanib for 4 weeks using the TRIzol reagent (15596026, Invitrogen). The subsequent RNA sequencing (RNA-seq) was performed by LC-Bio, and the data obtained were deposited in the NCBI Gene Expression Omnibus (GSE284661) database under a specific GSE accession number.

### Sulforhodamine B (SRB) staining colorimetry detects cell proliferation

Cell viability was determined using the sulforhodamine B (SRB; S1402, Sigma-Aldrich) assay with cells seeded in 96-well plates and incubated for 24 hours. After experimental treatments, cells were fixed with 10% cold trichloroacetic acid (T104257, Aladdin) for 3 hours at 4 °C, then washed and dried. SRB was added for 20 minutes, followed by washing with 0.1% glacial acetic acid and drying. The SRB was dissolved with tris-base (1115GR500, BioFroxx), and absorbance was read at 515 nm using SpectraMax M5/M5e Multimode Microplate Reader (Molecular Devices).

### Flow cytometry analysis of Annexin V-PI staining

The Apoptosis Assay Kit I (C1062L, Beyotime) was utilized to assess the rate of apoptosis following the manufacturer's instructions. After treatment, cells were harvested, washed with PBS, and stained with Annexin V and propidium iodide (PI). The BD FACSCalibur™ Flow Cytometer from BD Biosciences was used to measure 10,000 cells per sample. The staining results identified live cells as Annexin V-PI-, necrotic cells as Annexin V-PI+, early apoptotic cells as Annexin V+PI-, and late apoptotic cells as Annexin V+PI+.

### Mitochondrial membrane potential detected by flow cytometry

Mitochondrial membrane potential (MMP) was evaluated through JC-1 staining. After exposure to vandetanib for a set period, cells were harvested with trypsin and stained with JC-1 staining kit (C2005, Beyotime) for 30 minutes at 37°C, protected from light. The BD FACSCalibur™ Flow Cytometer was employed to measure MMP, with each sample analyzed by counting 10,000 cells.

### Quantitative reverse transcription polymerase chain reaction (qRT-PCR)

RNA was extracted from cells and tissues using Trizol reagent (15596026, Invitrogen). The RNA was subsequently reverse-transcribed into cDNA using a cDNA synthesis kit (AT311-02, TransGene Biotech). qRT-PCR was performed with the iTaq Universal SYBR Green Supermix (1725125, Bio-Rad) on a Thermal Cycler (Applied Biosystems). The PCR protocol included an initial denaturation at 95 °C for 3 minutes, followed by 40 cycles of 95 °C for 3 seconds and 60 °C for 31 seconds. The melting curves were examined for data analysis. Gene expression was quantified using the 2^^-ΔΔCt^ method, with each sample run in duplicate and each experiment repeated three times for accuracy. Primers for these experiments were both designed and synthesized by Youkang Biotechnology, with their sequences detailed in Supplementary Table 2.

### Western blot

Proteins were extracted from cell and liver samples using a RIPA Lysis (containing 50 mM Tris pH 7.4), 150 mM NaCl, 1% Triton X-100, 1% sodium deoxycholate, 0.1% SDS, 2 mM sodium pyrophosphate, 25 mM β-glycerophosphate, 1 mM EDTA). Protein lysates, 30-50 μg each, were loaded onto 8%, 10%, or 12% SDS-polyacrylamide gels for electrophoresis. The proteins were transferred to a PVDF membrane (Millipore Corporation) and incubated with primary antibodies for 16 h at 4 °C. After washing with T-PBS (PBS with 0.1% Tween-20) for a total of 25 minutes, the membranes were incubated with secondary antibodies for 1 hour at room temperature. Following three more washes with T-PBS, the membranes were developed using Western Lightning Plus-ECL reagent (P2300, NCM Biotech) as per the manufacturer's instructions.

Primary antibodies directed against cleaved-PARP (#94885S, 1:1000, Species reactivity: Human, Mouse), cleaved caspase-3 (#9664, 1:1000), CTSB (#31718, 1:1000), STAT3 (#9139,1:1000), AMPK (#2532, 1:1000), p-STAT3 (Tyr705) (#9145, 1:1000), p-AMPK (Thr172/183) (#50081, 1:2000) and ATG7 (#8558S, 1:1000) were purchased from Cell Signaling Technology. Primary antibodies directed against ACTB (db7283, 1:3000), GAPDH (db106, 1:10000), HA tag (db2603, 1:1000) and FLAG tag (db7002, 1:1000) were purchased from Diagbio. Primary antibodies directed against LC3 (M152-3, 1:2000) were purchased from MBL Beijing Biotech Co., Ltd. Primary antibodies directed against MCOLN1 (sc-398868, 1:500) were purchased from Santa Cruz Biotechnology. Primary antibody directed against cleaved-PARP (ET1608-10, 1:1000, Species reactivity: Human), CTSL (HA722063, 1:500), Lamin B1 (ET1606-27, 1:2000) and ATG5 (ET1611-38, 1:1000) were purchased from Huabio. Secondary antibodies conjugated with horseradish peroxidase (HRP), identified by catalog numbers PDR007 and PDM007 and used at a dilution of 1:1000, were sourced from Hangzhou Fude Biological technology CO., LTD. The RRIDs for all antibodies used in this study are comprehensively documented in Supplementary Table 1.

### ATP measurement

ATP levels were determined with the ATP Assay Kit (S0027, Beyotime). Culture medium was removed, and lysate was added for cell lysis at specified volumes. After centrifugation, the supernatant was taken for analysis. ATP assay mixture was prepared as per the instructions and added to the wells, equilibrating at room temperature for 3 to 5 minutes. Then, 20 μL of samples or standards were added, mixed, and their relative light units (RLU) were measured with a luminometer every 2 seconds or more. A series of ATP standard dilutions were made to establish a concentration gradient. The standard curve was generated according to the kit's guidelines. Using a SpectraMax M5/M5e Multimode Microplate Reader Molecular Device, RLU readings were recorded until the absorbance values became consistent.

### TUNEL staining

The tissue samples were fixed with formalin (F8775, Sigma-Aldrich), embedded in paraffin, and cut into 3-micrometer sections. Following deparaffinization and rehydration, the sections were exposed to a proteinase K solution (20 μg/mL in 10 mM Tris/HCl, pH 7.4-8.0) for 15 minutes at room temperature. The slides were rinsed with PBS three times. Subsequently, 50 μL of the TUNEL reaction mixture (C1088, Beyotime) was added and the sections were incubated for 60 minutes in a dark, humid chamber at 37 °C. After three more washes with PBS, the nuclei were stained with DAPI (D212, 1:5000, Dojindo). The immunofluorescent staining was then observed and imaged using a fluorescence microscope (Leica).

### Histological analysis

Mouse tissues were collected and fixed in a 10% phosphate-buffered formalin solution (with 137 mM NaCl, 2.7 mM KCl, 10 mM Na_2_HPO_4_, 1.76 mM K_2_HPO_4_, adjusted to pH 7.4) from Sigma-Aldrich (F8775). They were embedded in paraffin and sectioned at 5-micrometer intervals. After deparaffinization and hydration, the sections were stained with Hematoxylin and eosin, Sirius red, Masson's trichrome, or wheat germ agglutinin with Alexa FluorTM 488 conjugation (5 μg/mL, W11261, Thermo Scientific). The Hematoxylin and eosin, Sirius red, Masson's trichrome stained slides were imaged using the pathological section scanner HS6 (SUNNY INSTRUMENT) for and the wheat germ agglutinin were imaged by a fluorescence microscope (IX81-FV1000, Olympus).

### Immunohistochemical staining

Tissue paraffin sections underwent dewaxing, rehydration, and antigen retrieval, followed by a 10-minute treatment with 3% hydrogen peroxide (H_2_O_2_) (PV-6001, ZSGB-BIO) to inhibit endogenous peroxidase activity. After a 30-minute blocking period with 5% goat serum (16210064, Gibco), the sections were incubated with primary antibodies overnight at 4°C. These antibodies were then detected using horseradish peroxidase (HRP) conjugated secondary antibodies (PV-6001, PV-6002, ZSGB-BIO) for 60 minutes at room temperature. The slides were stained with a peroxidase substrate DAB kit (ZLI-9017, ZSGB-BIO), and the nuclei were lightly stained with hematoxylin (C0107, Beyotime) for 3 seconds. The immunostained sections were visualized and captured using a Leica light microscope. The primary antibodies used were specific for LC3B (#2775, 1:100), CTSB (#31718, 1:2500), cleaved caspase-3 (#9664, 1:100), p-STAT3 (Tyr705) (#9145, 1:100), p-AMPK (Thr172/183) (#50081, 1:200) and SQSTM1 (#88588S, 1:200), all purchased from Cell Signaling Technology.

### siRNA transfection

Cells were transfected with siRNA oligonucleotides using the oligofectamine™ reagent (12252011, Invitrogen) at a final concentration of 40 nM. The medium in a 6-well plate was replaced with a transfection mixture made with opti-MEM™ medium (31985070, Gibco). After a 6-hour incubation for transfection, the medium was removed and full growth medium was added. The cells then received the appropriate drug treatments according to the experimental design. Both the siRNA and its negative control (NC) were procured from Beijing Tsingke Biotech, with their sequences detailed in Supplementary table 3.

### Autophagy flux detection

Cells were grown in Nunc™ Lab-Tek™ II Chamber Slides™ (Thermo Fisher Scientific, 154534) and infected with an adenovirus encoding the fluorescent marker mCherry-GFP-LC3B, with subsequent treatments as indicated in the figure legends. They were fixed with 4% paraformaldehyde (P6148, Sigma-Aldrich) in PBS (10010023, Gibco) for 20 minutes at room temperature. After washing with PBS, cells were permeabilized with a cold 0.1% Triton X-100 solution (1139ML100, BioFROXX) for 10 minutes and stained with DAPI (D212, Dojindo) for 5 minutes. The cells were then mounted for analysis using a Leica confocal microscope. The number of cells exhibiting the specific mCherry-GFP-LC3B fluorescence was quantified using the Analyze Particles tool in ImageJ software.

### Echocardiography

Mice cardiac function was evaluated with the Vevo3100 Imaging System (Fujifilm Visual Sonics, Vevo3100) while under the effect of 1% isoflurane anesthesia (RWD Lifescience, R510-22). Measurements of left ventricular diameter in diastole and systole were taken using the Visual Sonics software, which also helped calculate the end-diastolic and end-systolic volumes. The left ventricular ejection fraction (LVEF) was calculated using the formula: LVEF (%) = [(end-diastolic volume - end-systolic volume) / end-diastolic volume] × 100. The left ventricular fractional shortening (LVFS) was determined with the formula: LVFS (%) = [(left ventricular diameter in diastole - diameter in systole) / left ventricular diameter in diastole] × 100.

### Immunofluorescence staining

Cells on slides were washed with PBS, fixed with 4% paraformaldehyde (P6148, Sigma-Aldrich) for 15 minutes, permeabilized with 0.3% Triton X-100 (1139ML100, Biofroxx) for 10 minutes, and blocked with 4% BSA (B2064, Sigma-Aldrich) in Tris-buffered saline for 30 minutes. They were then incubated with primary antibodies overnight at 4°C, followed by incubation with Alexa Fluor-conjugated secondary antibodies (Thermo Fisher Scientific, 1:200) for 1 hour at room temperature. After staining with DAPI (D212, 1:5000, Dojindo) for 5 minutes, the cells were mounted for fluorescence microscopy. The primary antibody STAT3 (#9139, 1:200), LC3B (#2775s, 1:200) and LAMP1 (#15665, 1:200) were purchased from Cell Signaling Technology. The primary antibody Galectin-3 (sc-23938, 1:200) was purchased from Santa Cruz Biotechnology. The signals were observed with fluorescence microscope (IX81-FV1000, Olympus).

### Nucleoplasmic dissociation

Nuclear and cytoplasmic fractions were isolated using the Nuclear/Cytosol Fractionation Kit (ab289882, Abcam). Liver tissues were minced before lysing, and HL-7702 cells were collected by centrifugation and resuspended in fractionation buffer at a ratio of 100 μL per 5 × 10^6^ cells. After centrifugation and collection of the supernatant, the nuclear pellet was lysed with RIPA buffer (P0013B, Beyotime). Both fractions were centrifuged at 16,000 g for 30 minutes at 4 °C, and the supernatants were taken for analysis. Samples were loaded with 2.5× sample buffer, and protein expression was assessed via western blot.

### Lyso-tracker staining

HL-7702 cells' lysosomal content was assessed using a lysosome-specific fluorescent probe (C1046, Beyotime). The cells were cultured on petri dishes. Once they reached the required confluence, the medium was aspirated, and a pre-warmed staining solution with 100 nM Lyso-Tracker was introduced for 25 minutes under cell culture conditions, followed by the addition of Hoechst33258 (C1011, Beyotime) to stain the nuclei for 5 minutes. The cells were visualized using a Leica fluorescence microscope.

### Fluo-4 AM staining

Intracellular calcium content of HL-7702 was assessed using a calcium ion fluorescent probe (C1060, Beyotime). Cells are cultured on Petri dishes. Once they reach the desired confluency, aspirate the medium and add a working solution containing 1 μM Fluo-4 AM and incubate at 37°C for 30 min, followed by Hoechst (C1011, Beyotime) to stain the nuclei for 5 min. The cells were observed using a Leica fluorescence microscope.

### Extraction of mouse primary hepatocytes

Primary hepatocytes from 6- to 8-week-old male C57BL/6J mice were isolated using a collagenase centrifugation and gradient method. The livers were infused with an intravenous collagenase solution (17104019, Sigma-Aldrich) that was dissolved in HBSS (14065056, Gibco). The cells were filtered through a 70-micrometer sieve. Then cells were purified by one-step density gradient centrifugation with percoll (P8370, Solarbio) and plated in petri dishes that had been precoated with type I collagen (C8065, Solarbio) for culture.

### Cellular thermal shift assay (CETSA)

The lysate of HEK-293T cells was treated with CA-074, apigenin, baicalein, or tannic acid at a final concentration of 10 μM and incubated for 30 minutes at room temperature. A control group received an equal volume of DMSO. The treated samples were divided into twelve parts and subjected to incubation at a range of temperatures (42.0 ℃, 42.5 ℃, 43.7 ℃, 45.6 ℃, 48.2 ℃, 50.8 ℃, 53.2 ℃, 55.8 ℃, 58.4 ℃, 60.3 ℃, 61.5 ℃, and 62.0 ℃) for 6 minutes each. After the temperature gradient incubation, the samples were centrifuged at 12,000 rpm for 15 minutes. The expression of the protein cathepsin B (CTSB) in the supernatant was then analyzed using SDS-PAGE.

### CTSB protease assays

Cathepsin B (CTSB) enzymatic activity was measured using the fluorogenic substrate Z-Arg-Arg-AM Chydrochloride (HY-134434, MedChemExpress). The substrate was prepared in 2× reaction buffer (25 mM Tris-HCl pH 8.0, 100 mM NaCl, 10% glycerol, 0.8 mM sodium acetate pH 6.0, 8 mM EDTA). Reactions contained 100 ng recombinant CTSB (TMPY-00731, TargetMol), 10 μM substrate, and 1 mM test compound in a final volume of 20 μL, incubated at 37°C for 60 min. Fluorescence was quantified on a SpectraMax M5/M5e Multimode Microplate Reader (Molecular Devices) at 380 nm excitation/460 nm emission. Background signal was corrected using distilled deionized water. Percentage inhibition was calculated as: [1 - (fluorescence with compound / fluorescence of control)] × 100%.

### Transmission electron microscope (TEM)

Mouse tissues, about 1 mm³ in size, were fixed in a 2.5% glutaraldehyde solution (G5882, Sigma-Aldrich) with a pH of 7.2 at room temperature for 2 h and stored at 4°C overnight. Tissue blocks were fixed with 1% osmic acid for 1 hour and stained with 2% uranium for 0.5 hours. After dehydration and embedding, the tissues were sectioned with an ultrathin slicer. The ultrathin sections were examined under an electron microscope (H7650, Hitachi).

### Computer simulation of molecular docking techniques

The crystal structure for the enzyme cathepsin B (CTSB, with the Protein Data Bank identification number 8b4t) was retrieved from the Protein Data Bank. Additionally, the structure for tannic acid was sourced from the PubChem database using the compound's identifier (PubChem CID 16129778). The docking of CTSB with tannic acid was performed utilizing AutoDockTools version 1.5.6, with the docking parameters configured according to the methodology detailed by Ferreira LG (Ferreira et al. 2015). The optimal outcomes of the docking simulations were then visualized employing PyMOL software.

### Plasmid construction and transfection

The cDNA of CTSB was amplified from the reverse-transcribed RNA and then subcloned into the pcDNA3.0-CTSB-Flag vector. The pcDNA3.0-CTSB-Flag construct was created through restriction enzyme digestion using BamHI (R3136V, NEB) and XhoI (R0146V, NEB). One-site mutant plasmids of pcDNA3.0-AMPK-T183D-HA was generated by using Hieff Mut™ Site-Directed Mutagenesis Kit (Yeasen, 11003ES10). Cells were transfected using Lipofectamine 2000 (11668019, Invitrogen) following the manufacturer's protocol. In brief, cells were seeded and allowed to reach approximately 70% confluence. The relevant plasmid was then introduced into the cells along with the transfection reagent for a period of 4-6 hours, after which the medium was replaced with fresh culture medium.

### Statistical analysis

Results are shown as averages with means ± standard deviations (SDs). Significance was considered when the p value dropped below 0.05. For pair-wise group comparisons, the student's *t* test was implemented, and for assessing differences among multiple groups, a one-way ANOVA was conducted. Statistical processing was handled with Microsoft Excel (edition 1808), ImageJ (edition 1.8.0), and GraphPad Prism Software (edition 8.0). Detailed statistical information, such as the specific number of mice utilized, is delineated in the Figure legends for each study. No data points were omitted from the analyses.

## Results

### Vandetanib induces hepato-cardiotoxicity and apoptosis

**Figure 1:**
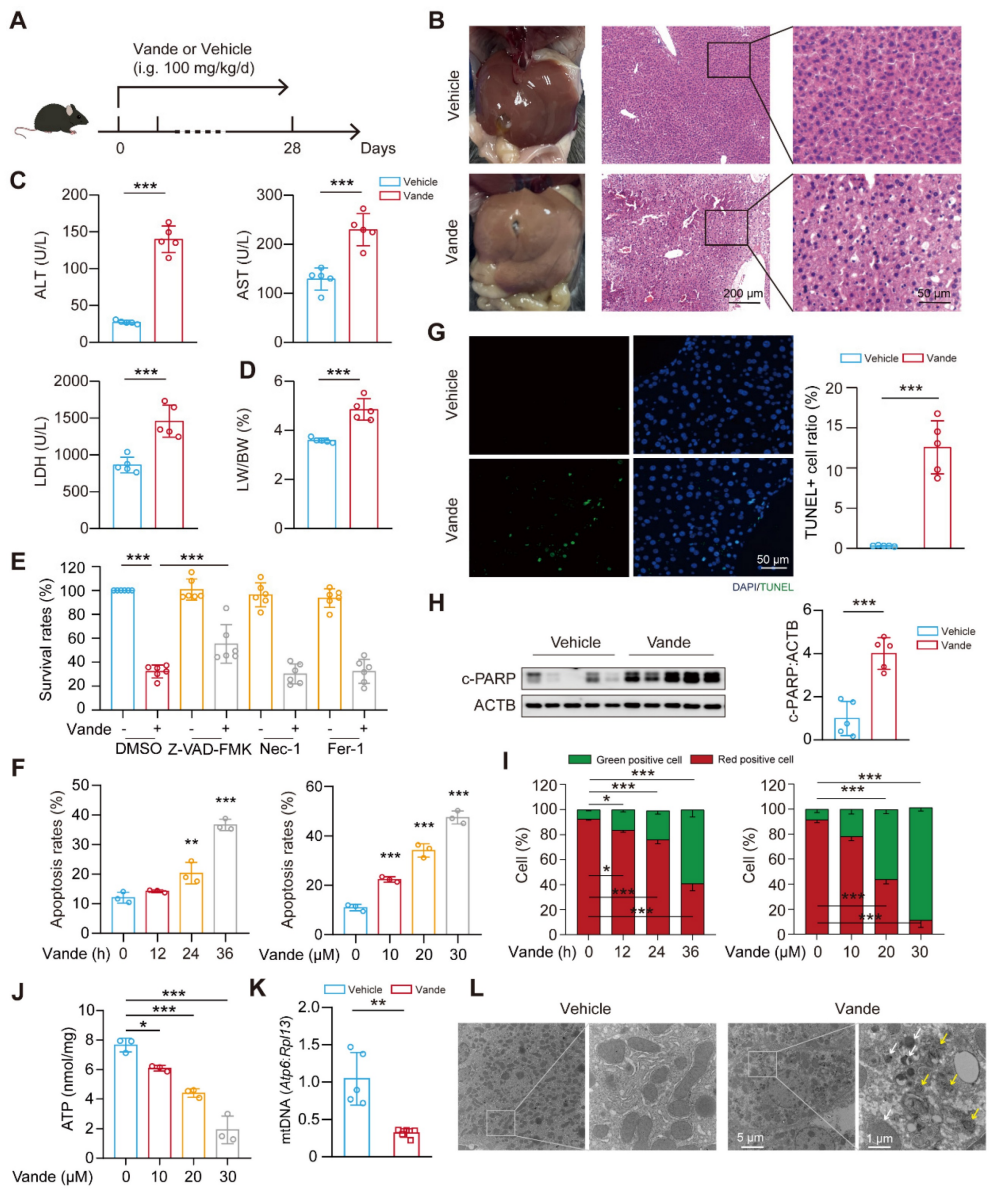
** Vandetanib induced hepatotoxicity and mitochondria-dependent apoptosis in hepatocytes.** (**A**) Mouse model construction of vandetanib-induced toxicity. C57BL/6J mice were treated with 0.5% CMC-Na or 100 mg/kg/day vandetanib for 4 weeks. (**B**) (Left panel) Representative images of liver tissues. (Right panel) Representative images of H&E staining in liver tissues. For 50× magnification, scale bar, 200 µm; for 200× magnification, scale bar, 50 µm. (**C**) ALT, AST and LDH levels of mice. (n = 5). (**D**) The liver weight to body weight ratio (LW/BW) of mice. (n = 5). (**E**) HL-7702 cells were treated with 20 μM vandetanib and/or 20 μM Z-VAD-FMK, 20 μM Nec-1, 10 μM Fer-1 for 48 h. (n = 6). (**F**) HL-7702 cells were treated with 20 μM vandetanib for 0, 12, 24 or 36 h or 0, 10, 20 or 30 μM vandetanib for 36 h. Then, the apoptosis rates were detected by flow cytometry with Annexin V/PI staining. (n = 3). (**G**) Representative images of TUNEL staining in liver tissues. Scale bar, 50 μm. (**H**) The expression levels of c-PARP in liver tissues were measured by western blot. (n = 5). (**I**) HL-7702 cells were treated with 20 μM vandetanib for 0, 12, 24 or 36 h or 0, 10, 20, 30 μM vandetanib for 36 h. Then, cells were harvested and stained with JC-1. The mitochondrial membrane potential was detected by flow cytometry. (n = 3). (**J**) The ATP levels in HL-7702 cells treated with 0, 10, 20, 30 μM vandetanib for 36 h were detected. (n = 3). (**K**) The relative mitochondrial DNA (mtDNA) levels in liver tissues. (n = 5). (**L**) Transmission electron microscopy observation of the liver tissues. Representative images of mitochondrial structure were shown on the left (scale bar, 5 μm) and enlarged view on the right (scale bar, 1 μm). The yellow arrows: abnormal membrane structure organelles; the white arrows: damaged mitochondria. Data are represented as the mean ± SD. *p < 0.05, **p < 0.01, ***p < 0.001. Unpaired two-sided Student's *t* test for (**C**), (**D**), (**G**), (**H**) and (**K**). One way ANOVA followed by Tukey post hoc test for (**E**). One way ANOVA followed by Dunnett T3 post hoc test for (**F**), (**I**), (**J**).

Hepatotoxicity constitutes a major clinical challenge during therapeutic deployment of TKIs in oncology. To systematically investigate the molecular mechanisms underlying vandetanib-induced hepatotoxicity in the clinic, C57BL/6J mice received a four-week oral gavage of 0.5% CMC-Na or vandetanib (at dosages of 100 mg/kg, about 2-folds of clinical doses) (**Fig. 1A**). Hematoxylin and eosin (H&E) staining revealed that hepatocytes of vandetanib-treated mice exhibited blurred borders, severe vacuolization, and nuclear shrinkage (**Fig. 1B**). Alanine aminotransferase (ALT), aspartate aminotransferase (AST), and lactate dehydrogenase (LDH) levels had a marked increase following vandetanib administration (**Fig. 1C**). Concurrently, hepatosomatic index (liver-to-body weight ratio) measurements showed a statistically significant increase relative to the control group (**Fig. 1D**). Given the FDA's black box warning for vandetanib due to severe and life-threatening cardiotoxicity, we also investigated its cardiotoxic effects. Echocardiographic assessments conducted on mice revealed a notable decrease in left ventricular ejection fraction (LVEF) and fractional shortening (LVFS) ratios following the administration of vandetanib (**Supplementary Fig. 1A, B**). ​**​**Cardiac morphological alterations were significantly induced by vandetanib relative to controls. This result is consistent with the results of heart weight ratio (HW/BW) and heart tibia ratio (HW/HL) (**Supplementary Fig. 1C, D**). H&E staining showed that the cardiac tissue sections exhibited cytoplasmic dispersion, cellular vacuolization, and decreased myocardial cell density after vandetanib treatment (**Supplementary Fig. 1E**). When cardiac contractile dysfunction occurs, it is frequently associated with remodeling changes^23^. Wheat germ agglutinin (WGA) staining demonstrated a significant increase in myocardial cross-sectional area following vandetanib administration (**Supplementary Fig. 1F**), aligning with the findings from the patient's autopsy^3^. qRT-PCR analysis indicated that the administration of vandetanib results in a significant up-regulation of *Myh7*, *Nppa*, and *Nppb* expression levels, accompanied by a notable down-regulation of *Myh6*, thereby suggesting that vandetanib treatment induces compensatory remodeling in the cardiac tissue (**Supplementary Fig. 1G**). These findings conclusively demonstrate the hepato-cardiotoxic effect of vandetanib in murine models.

Next, we investigated the mechanism underlying hepatocyte injury induced by vandetanib. First, we evaluated the survival rates of human hepatocytes which were significantly reduced upon vandetanib treatment (**Supplementary Fig. 2A, B**). To delineate the predominant cell death modality, hepatocytes were co-treated with vandetanib and an apoptosis inhibitor Z-VAD-FMK, a necrosis inhibitor Nec-1, and a ferroptosis inhibitor Fer-1. The results indicated that only Z-VAD-FMK effectively protected hepatocytes from the survival rate reduction caused by vandetanib (**Fig. 1E, Supplementary Fig. 2C**), implicating apoptotic pathways in vandetanib-mediated hepatotoxicity. Furthermore, immunoblot analyses confirmed marked upregulation of cleaved-poly (ADP-ribose) polymerase (c-PARP) expression in both *in vivo* (**Supplementary Fig. 2D**), post-vandetanib treatment. PI/Annexin V staining with flow cytometry analysis revealed that vandetanib-induced hepatocyte apoptosis in a dose- and time-dependent manner (**Fig. 1F**). Notably, the apoptotic response was significantly attenuated upon Z-VAD-FMK co-administration (**Supplementary Fig. 2E, F**). Consistent with these findings, TUNEL (terminal deoxynucleotidyl transferase dUTP nick end labeling) assay revealed pronounced nuclear DNA fragmentation within hepatic parenchyma, a hallmark of late-stage apoptosis, following vandetanib administration, as evidenced by a notable colocalization of green fluorescence with the nuclear region of hepatocytes (**Fig. 1G**). Vandetanib administration also significantly elevated c-PARP levels in murine hepatic tissues (**Fig. 1H**). Similarly, both *in vitro* and *in vivo* experiments demonstrated vandetanib-induced apoptosis in cardiomyocytes, further confirming its cardiotoxic effects (**Supplementary Fig. 2G, H**). Together, these multimodal experimental approaches demonstrate that vandetanib induces hepatocyte and cardiomyocyte apoptosis both *in vivo* and* in vitro*.

Altered mitochondrial membrane potential (MMP) dissipation represents a critical initiating event in apoptotic cascades, and thus, we performed JC-1 staining followed by flow cytometry to detect the MMP. The results showed that vandetanib decreased MMP in a dose- and time-dependent manner (**Fig. 1I**), suggesting that vandetanib may induce hepatocyte apoptosis by impairing mitochondrial function. Subsequently, we further evaluated the effects of vandetanib on mitochondria by detecting the levels of ATP and mitochondrial DNA (mtDNA) (**Fig. 1J, K**). Both the severely reduced copy number of mtDNA and impaired mitochondrial ATP synthesis capacity indicate that vandetanib-induced liver damage occurs through the induction of mitochondria-dependent apoptosis. Transmission electron microscopy (TEM) analysis revealed marked mitochondrial fragmentation and proliferation of aberrant membranous structures within hepatocytes from vandetanib-treated mice (**Fig. 1L**), suggesting mitochondria-dependent apoptotic activation, with these ultrastructural alterations potentially constituting a principal hepatotoxic mechanism.

### Vandetanib induces CTSB accumulation by activating STAT3

**Figure 2:**
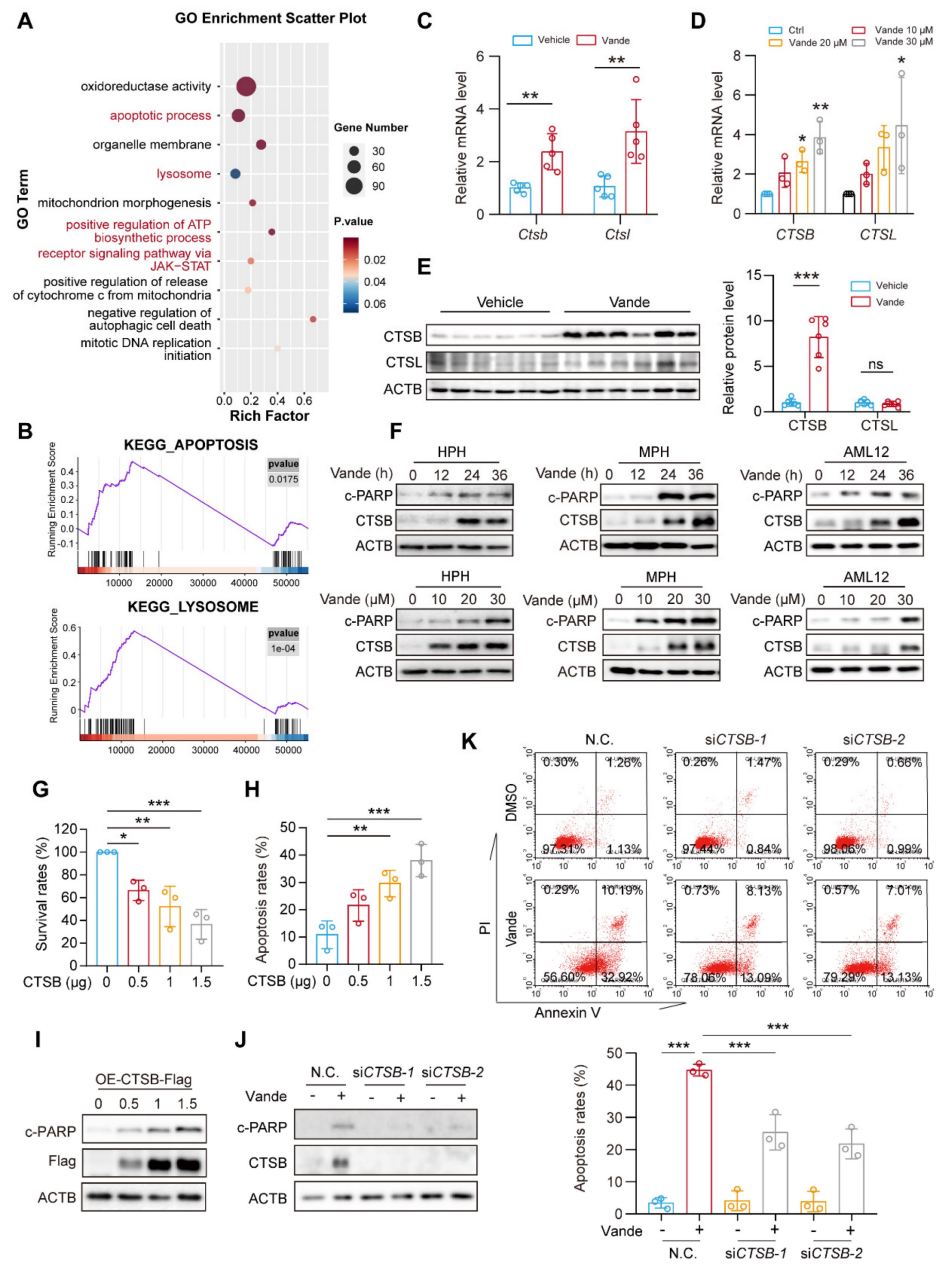
** Vandetanib induced hepatocyte apoptosis by upregulating CTSB.** (**A**) GO enrichment analysis of alterable protein expression after vandetanib's treatment. The red font highlighted parts mainly divided into apoptosis and lysosome pathways. (**B**) GSEA plots for apoptosis and lysosome KEGG pathways significantly enriched after vandetanib's treatment. (**C**) The mRNA levels of *Ctsb* and *Ctsl* in liver tissues were measured by qRT-PCR. (n = 5). (**D**) HL-7702 cells were treated with 0, 10, 20 and 30 μM vandetanib for 24 h. The mRNA levels of *CTSB* and *CTSL* were measured by qRT-PCR. (n = 3). (**E**) The protein levels of CTSB and CTSL in liver lysates were measured by western blot. (n = 6). (**F**) Human primary hepatocytes (HPH), murine primary hepatocytes (MPH), and AML12 cells were treated with 20 μM vandetanib for 0, 12, 24, 36 h or 0, 10, 20, 30 μM vandetanib for 36 h. The expression levels of c-PARP and CTSB were measured by western blot. (**G-I**) HL-7702 cells were transfected with vector, 0.5, 1 or 1.5 μg pcDNA3.0-CTSB-Flag plasmid for 36 h. (**G**) The survival rates were measured by SRB staining. (n = 3). (**H**) The apoptosis rates were detected by flow cytometry of Annexin V/PI staining. (n = 3). (**I**) The expression levels of c-PARP in total cell lysates were detected by western blot. (**J-K**) HL-7702 cells were transfected with negative control or siRNA targeting *CTSB*, and then treated with or without 20 μM vandetanib for 36 h. (**J**) The expression levels of c-PARP and CTSB in HL-7702 cells were measured by western blot. (**K**) The apoptosis rates were detected by flow cytometry of Annexin V/PI staining. Data are represented as the mean ± SD. ns, no significance, *p < 0.05, **p < 0.01, ***p < 0.001. Unpaired two-sided Student's *t* test for (**C**) and (**E**). One way ANOVA followed by Dunnett T3 post hoc test for (**D**), (**G**) and (**H**). One way ANOVA followed by Tukey post hoc test for (**K**).

As a kinase inhibitor, vandetanib perturbs cellular signaling pathways that may significantly contribute to its toxic effects. To systematically identify molecular determinants of vandetanib-induced hepatotoxicity, we performed RNA sequencing (RNA-Seq) analysis. Gene Ontology (GO) enrichment analysis revealed significant pathway activation in apoptotic, lysosomal, and ATP synthesis within vandetanib-treated murine hepatic tissue (**Fig. 2A**). Complementary Gene Set Enrichment Analysis (GSEA) enrichment analysis confirmed strong concordance in apoptotic process and lysosomal pathway activation, indicating lysosomal dysregulation as a pivotal driver of vandetanib-induced hepatocyte apoptosis and hepatotoxicity (**Fig. 2B**). Transcriptomic profiling prioritized CTSB and CTSL as candidate mediators of vandetanib hepatotoxicity based on their significant transcriptional upregulation post-treatment. These two lysosomal proteases, which mediate diverse cellular processes through lysosomal regulation, exhibit well-characterized mechanistic associations with multiple pathologies as documented in prior mechanistic studies 24, 25. Consistent with transcriptomic profiling data, the expression levels of *Ctsb* and *Ctsl* significantly upregulated *in vivo* and *in vitro* (**Fig. 2C, D**). Immunoblot analyses confirmed substantial upregulation of CTSB protein *in vivo,* whereas CTSL protein levels remained unchanged despite transcriptional activation (**Fig. 2E**). As for the reason why the protein level of CTSL remained unchanged, we speculate that it may be related to its translation synthesis and protein homeostasis regulation. Multispecies validation across human primary hepatocytes (HPH), murine primary hepatocytes (MPH), and mouse hepatocyte cell line AML12 hepatocyte lineages demonstrated concomitant elevation of c-PARP and CTSB protein levels following vandetanib exposure (**Fig. 2F**). In addition, KEGG pathway analysis of differentially expressed genes identified significant enrichment of apoptosis-related pathways and JAK-STAT signaling activation (**Supplementary Fig. 3A, B**). STAT3, a member of the STAT family, plays a pivotal role in the pathogenesis of liver diseases. Aberrant expression of various acute phase proteins, chemokines, and chemokine receptors due to activated STAT3 can aggravate liver inflammation in cases of CCl_4_-induced liver fibrosis 26. STAT3 has been reported to regulate the transcriptional levels of *Ctsb* and *Ctsl*^27^. The phosphorylation of STAT3 at Tyr705 is essential for its transcriptional regulatory function, which enables STAT3 to translocate into the nucleus. Following the administration of vandetanib, immunofluorescence analysis revealed an increase in fluorescence intensity, which indicates the nuclear translocation of STAT3 (**Supplementary Fig. 3C**). Subsequently, we isolated the cytoplasmic and nuclear components to evaluate the distribution of STAT3. The results demonstrated a significant increase in the nuclear localization of STAT3 in hepatocytes following treatment with vandetanib (**Supplementary Fig. 3D**). Furthermore both *in vitro* and *in vivo* data demonstrated increased phosphorylation of STAT3 (Tyr705) (**Supplementary Fig. 3E-G**). Importantly, STAT3-IN-13, a specific inhibitor of p-STAT3 (Tyr705), effectively reversed vandetanib-induced transcriptional upregulation of *Ctsb* (**Supplementary Fig. 3H**). Collectively, these results indicate that vandetanib upregulates CTSB expression through STAT3 phosphorylation at Tyr705.

**​​**

### ​​CTSB plays a key role in vandetanib-induced hepato-cardiotoxicity

The expression of CTSB is widely observed in liver biopsy tissues from patients with various liver diseases, and elevated CTSB is believed to be closely associated with liver fibrosis and subsequent cirrhosis 28. To evaluate the role of CTSB in vandetanib-induced liver injury, we transfected the HL-7702 cell line with a plasmid that overexpresses CTSB to simulate elevated levels of this protein. The results revealed that CTSB overexpression inhibits cell viability in a dose-dependent manner (**Fig. 2G**). Additionally, PI/Annexin V staining followed by flow cytometry analysis indicated that the exogenous overexpression of CTSB enhances apoptosis in HL-7702 cells, a finding further confirmed by western blotting (**Fig. 2H, I**). To further establish that the hepatocyte apoptosis induced by vandetanib is linked to the upregulation of CTSB, we employed two siRNA sequences to genetically knock down CTSB. This knockdown resulted in the reversal of apoptosis (**Fig. 2J, K**), while silencing of *CTSL* did not impact vandetanib-induced apoptosis (**Supplementary Fig. 4**).

Building on these hepatic findings, we next investigated whether CTSB also mediates vandetanib-induced cardiotoxicity​​. Vandetanib treatment significantly upregulated CTSB levels *in vitro* and *in vivo* (**Supplementary Fig. 5A, B**). Critically, CTSB knockdown in cardiomyocytes attenuated vandetanib-induced apoptosis, whereas CTSB overexpression in primary cardiomyocytes directly triggered apoptotic cell death (**Supplementary Fig. 5C, D**).

Collectively, these data demonstrate that CTSB serves as a central mediator of vandetanib-induced hepato-cardiotoxicity, with its elevated levels directly driving apoptotic pathways in both hepatocytes and cardiomyocytes.

### Vandetanib triggers hepatotoxicity via CTSB-mediated lysosomal dysfunction and impaired AMPK signaling

**Figure 3:**
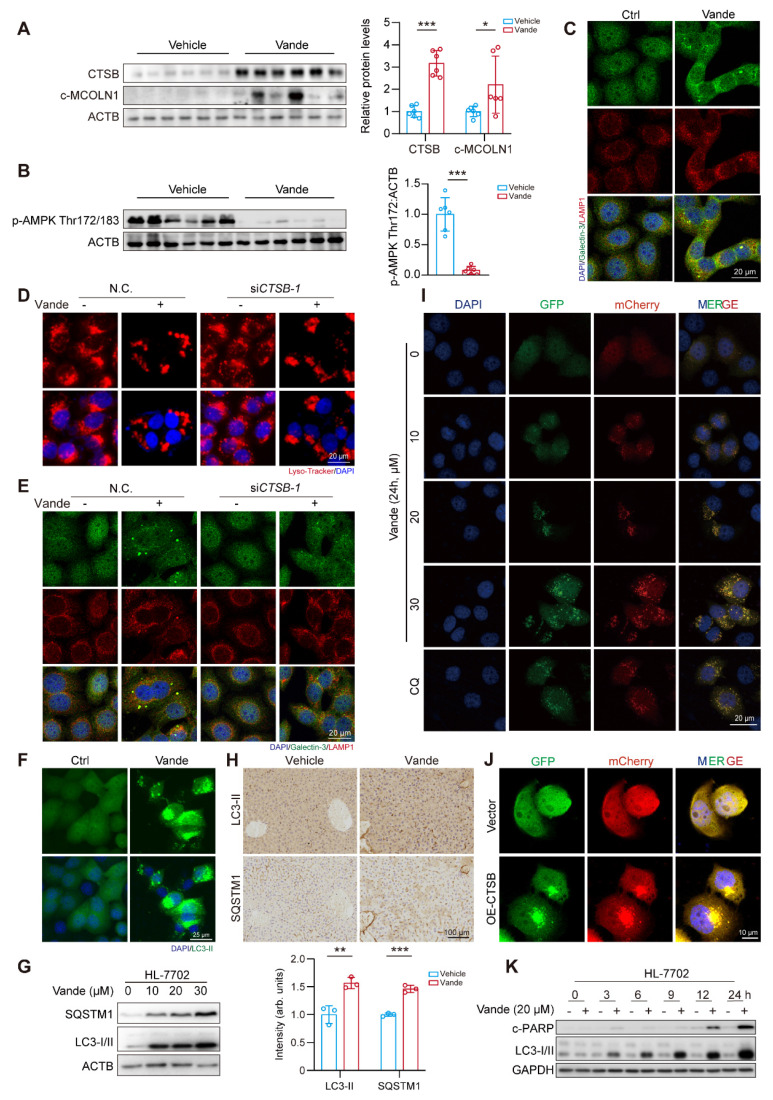
** Vandetanib induced lysosomal damage via CTSB-mediated cleavage of MCOLN1.** (**A**) The expression levels of CTSB and c-MCOLN1 in liver tissues of mice. (n = 6). (**B**) The expression levels of p-AMPK Thr172/183 in liver tissues of mice. (n = 6). (**C**) The expression levels of Galectin-3 and LAMP1 in HL-7702 cells treated with 20 μM vandetanib for 36 h were measured by immunofluorescence. Scale bar, 20 μm. (**D-E**) HL-7702 cells were transfected with negative control or siRNA targeting *CTSB*, and then treated with 20 μM vandetanib for 36 h. (**D**) Representative images of Lyso-Tracker staining in HL-7702. Scale bar, 20 μm. (**E**) The expression levels of Galectin-3 and LAMP1 in HL-7702 cells were measured by immunofluorescence. Scale bar, 20 μm. (**F**) HL-7702 cells were treated with 20 μM vandetanib for 36 h. The expression levels of LC3-II in HL-7702 cells were measured by immunofluorescence. Scale bar, 25 μm. (**G**) The expression levels of SQSTM1 and LC3-I/II in HL-7702 cells treated with 20 μM vandetanib for 0, 10, 20, 30 μM vandetanib for 36 h were detected by western blot. (**H**) The expression levels of LC3-II and SQSTM1 in liver tissues of mice were detected by immunohistochemical analysis. Scale bar, 100 μm. (**I**) Representative confocal fluorescence micrographs of HL-7702 cells transfected with Ad-mCherry-GFP-LC3B and treated with 0, 10, 20, 30 μM vandetanib for 24 h. Scale bar, 20 μm. (**J**) The expression levels of c-PARP and LC3-I/II in HL-7702 cells treated with 20 μM vandetanib for 0, 3, 6, 9, 12 and 24 h were detected by western blot. Data are represented as the mean ± SD. *p < 0.05, **p < 0.01, ***p < 0.001. Unpaired two-sided Student's *t* test for (**A**), (**B**) and (**H**).

As a pivotal lysosomal hydrolase, CTSB exerts regulatory control over lysosomal physiology through its proteolytic activity. Previous mechanistic studies established that CTSB-mediated cleavage of MCOLN1/TRPML1 calcium channels disrupts calcium homeostasis and lysosomal integrity 29. Our results showed that both vandetanib treatment and CTSB overexpression led to a significant accumulation of calcium ions within lysosomes (**Supplementary Fig. 6A, B**), an effect comparable to that observed with MCOLN1 knockdown alone (**Supplementary Fig. 6C**). Furthermore, GSEA also revealed a marked enrichment of calcium signaling pathways in vandetanib-treated hepatocytes. These findings suggest that vandetanib-induced dysregulation of calcium homeostasis, potentially mediated by the upregulation of CTSB, may play an important role in vandetanib-induced hepatotoxicity. AMPK can be regulated by calcium ion flux, altering the phosphorylation status of its Thr172/183 site, which is critical for liver homeostasis 30. Thus, we firstly examined changes in cleaved-MCOLN1 (c-MCOLN1) following vandetanib treatment and found a significant increase in c-MCOLN1 (**Fig. 3A**). Following treatment with vandetanib, p-AMPK (Thr172/183) in mouse liver was downregulated significantly (**Fig. 3B**). Additionally, overexpressing CTSB or knockdown of MCOLN1 produced similar results (**Supplementary Fig. 6D**). To further determine the role of p-AMPK (Thr183) in vandetanib-induced hepatotoxicity, we mutated threonine 183 of AMPK to aspartic acid (designated as AMPK-T183D) to simulate a state of continuous phosphorylation activation. Under vandetanib treatment, AMPK-T183D transfection partially recovered vandetanib-induced hepatocyte apoptosis and mitochondrial dysfunction (**Supplementary Fig. 7A-C**). Meanwhile, using metformin to restore AMPK Thr172/183 phosphorylation also reduced vandetanib-induced apoptosis in hepatocytes (**Supplementary Fig. 7D, E**). These results suggest that the mitochondria-dependent apoptosis in hepatocytes induced by vandetanib may be attributed to the inhibition of AMPK Thr172/183.

Lysosomes dysfunction is often reflected in changes in their morphology. Thus, we used LAMP1 immunofluorescence staining and Lyso-Tracker staining to detect the effect of vandetanib on lysosomes. The results revealed that treatment with vandetanib resulted in abnormal aggregation and enlargement of the lysosomes (**Supplementary Fig. 8A, B**). Additionally, there was an observable increase in the co-localization of Galectin-3, a known marker for the repair of damaged lysosomes, providing further evidence that vandetanib caused lysosomal damage (**Fig. 3C**). However, lysosomal functional abnormalities were restored after knockdown of CTSB in HL-7702 cells (**Fig. 3D, E**). These findings demonstrated vandetanib-induced hepatocyte lysosomal dysfunction through CTSB upregulation.

In conclusion, these results demonstrated that upregulation of CTSB plays a critical role in mitochondria-dependent hepatocyte apoptosis induced by vandetanib, as it leads to lysosomal damage through the cleavage of MCOLN1, subsequently disrupting calcium ion flux and inhibiting the phosphorylation of AMPK Thr172/183.

### Upregulation of CTSB causes hepatotoxicity by inducing autophagy inhibition

Autophagy functions as a dual regulator of cellular mechanisms, influencing cellular fate while serving as a signaling hub for promoting either cell survival or cell death under various conditions 31-34. Notably, lysosomal damage can impede the autophagic process. In light of these findings, we sought to investigate the potential involvement of autophagy in the hepatotoxicity induced by vandetanib. Initially, we assessed the expression levels of autophagy marker proteins, LC3-II and SQSTM1. Following treatment with vandetanib, both LC3-II and SQSTM1 exhibited significant upregulation in both *in vitro* and *in vivo* (**Fig. 3F-H**)*.* Subsequently, we infected hepatocytes with Ad-mCherry-GFP-LC3B to observe autophagic flux. Upon fusion of autophagosomes with lysosomes, the acidic environment within the lysosomes results in the quenching of GFP fluorescence, indicating the progression of autophagic flux.

**Figure 4:**
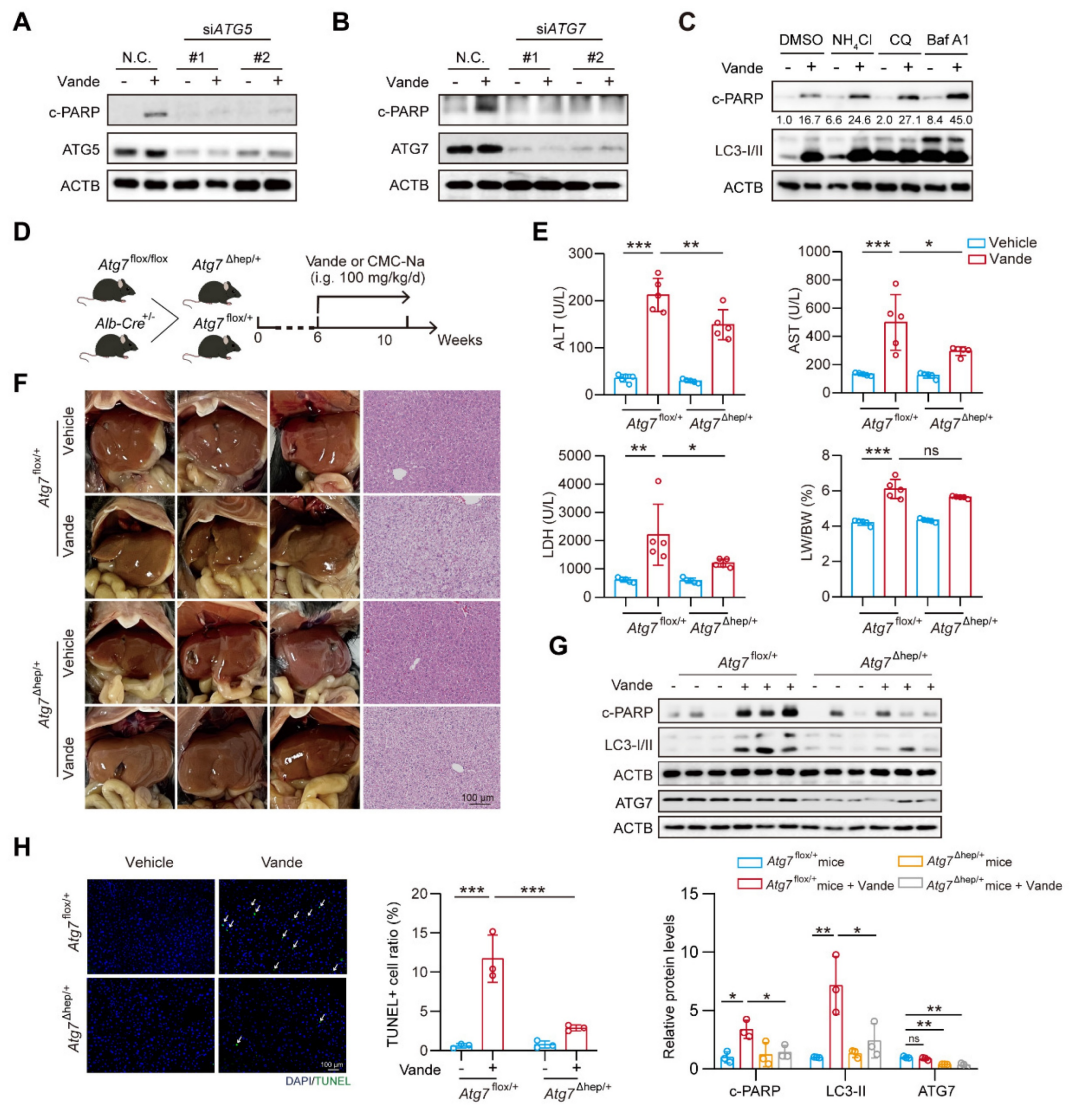
** Inhibition of autophagy alleviated vandetanib-induced hepatotoxicity​.** (**A**) HL-7702 cells were transfected with negative control or siRNA targeting *ATG5*, followed by treatment with 20 μM vandetanib for 36 h. The expression levels of c-PARP and ATG5 were detected by western blot. (**B**) HL-7702 cells were transfected with negative control or siRNA targeting *ATG7*, followed by treatment with 20 μM vandetanib for 36 h. The expression levels of c-PARP and ATG7 were detected by western blot. (**C**) HL-7702 cells were treated with 20 μM vandetanib and/or 5 mM NH_4_Cl, 20 μM CQ, 1 nM Bafilomycin A1 for 36 h. The expression levels of c-PARP and LC3-I/II were detected by western blot. (**D-H**) All mice were randomly divided into four groups as follows: the *Atg7*^flox/+^ or* Atg7*^Δhep/+^ group were treated with 0.5% CMC-Na or 100 mg/kg/day vandetanib for 4 weeks. (**D**) Schematic diagram of the experimental protocol for liver-specific *Atg7* knockout mice. (**E**) The ALT, AST, LDH levels of mice and the liver weight to body weight ratio (LW/BW) of mice. (n = 5). (**F**) (Left panel) Representative images of liver tissues. (Right panel) Representative images of H&E staining of liver tissues. Scale bar, 100 μm. (**G**) The expression levels of c-PARP, LC3-I/II and ATG7 were detected by western blot. (n = 3). (**H**) Representative images of TUNEL staining in liver tissues. Scale bar, 100 μm. Data are represented as the mean ± SD. ns, no significance, *p < 0.05, **p < 0.01, ***p < 0.001. One way ANOVA followed by Tukey post hoc test for (**E**), (**G**) and (**H**).

The results showed that, following treatment with vandetanib, the green fluorescence signal of GFP was not quenched and co-localized with mCherry to form a yellow signal, consistent with the autophagy inhibition observed in the positive control chloroquine (CQ), thereby suggesting that vandetanib blocks the fusion of autophagosomes and lysosomes, ultimately leading to autophagy inhibition (**Fig. 3I**). Then we examined the effect of overexpression of CTSB on lysosome and autophagic flux. The results indicated that the overexpression of CTSB produced a phenotype comparable to that observed with vandetanib treatment (**Fig. 3J**, **Supplementary Fig. 9**). To delve into the role of autophagy inhibition in vandetanib-induced apoptosis in hepatocytes, we examined the changes of LC3-I/II and c-PARP proteins over the time course. The results showed that vandetanib-induced LC3-II accumulation preceded apoptosis (**Fig. 3K**). Collectively, these results suggest that vandetanib inhibits the fusion of autophagosomes and lysosomes by impairing lysosomal function through upregulation of CTSB expression. To assess whether vandetanib-induced autophagy inhibition led to apoptosis in hepatocytes, we initially sought to ascertain the direct effect of vandetanib-induced autophagy on hepatocyte apoptosis. We utilized siRNA to selectively silence the expression of two proteins that are crucial for the early stages of autophagy, autophagy-associated proteins 5 (ATG5) and autophagy-related protein 7 (ATG7), to inhibit the initiation stage of autophagy. The results showed that silencing *ATG5* and *ATG7* led to decreased expression of c-PARP protein, indicating that inhibiting the initiation stage of autophagy partially alleviate the cell apoptosis induced by vandetanib (**Fig. 4A, B**). PI/Annexin V staining followed by flow cytometry showed that silencing *ATG5* or *ATG7* partially reduce the rate of vandetanib-induced apoptosis (**Supplementary Fig. 10A, B**). Furthermore, we evaluated the impact of inhibiting the fusion of autophagosomes and lysosomes on the vandetanib-induced apoptosis in hepatocytes. The results showed that autophagy late-stage inhibitors, including chloroquine (CQ), Bafilomycin A1 (Baf A1), and NH_4_Cl, significantly augment the apoptotic effects of vandetanib (**Fig. 4C**), thereby indicating that the inhibition of autophagosome-lysosome fusion may further exacerbate hepatocyte damage associated with vandetanib treatment.

Next, we developed a mouse model featuring liver-specific knockdown of the *Atg7* gene to further investigate the effects of inhibiting autophagy initiation on vandetanib-induced hepatotoxicity (**Fig. 4D**). The results showed that serum levels of ALT, AST, and LDH in *Atg7*^Δhep/+^ mice exhibited a partial decrease in comparison to *Atg7*^flox/+^ controls following treatment with vandetanib; however, there was no statistically significant alteration in the liver weight ratio (**Fig. 4E**). Additionally, H&E staining showed that specific knockdown of *Atg7* in mouse liver partially restored the hepatocyte nuclear shrinkage and vacuolization induced by vandetanib (**Fig. 4F**). Western blot and TUNEL staining indicated that the hepatocyte apoptosis induced by vandetanib was effectively reversed following the liver-specific knockdown of *Atg7* (**Fig. 4G, H**).

The results mentioned above collectively indicate that autophagy plays a role in vandetanib-induced liver injury. Inhibition of the initiation phase of autophagy may partially attenuate the vandetanib-induced hepatotoxic effects by reducing the generation of autophagosomes to alleviate the stress on damaged lysosomes.

### Knockdown of CTSB ameliorates vandetanib-induced hepatotoxicity *in vivo*

**Figure 5:**
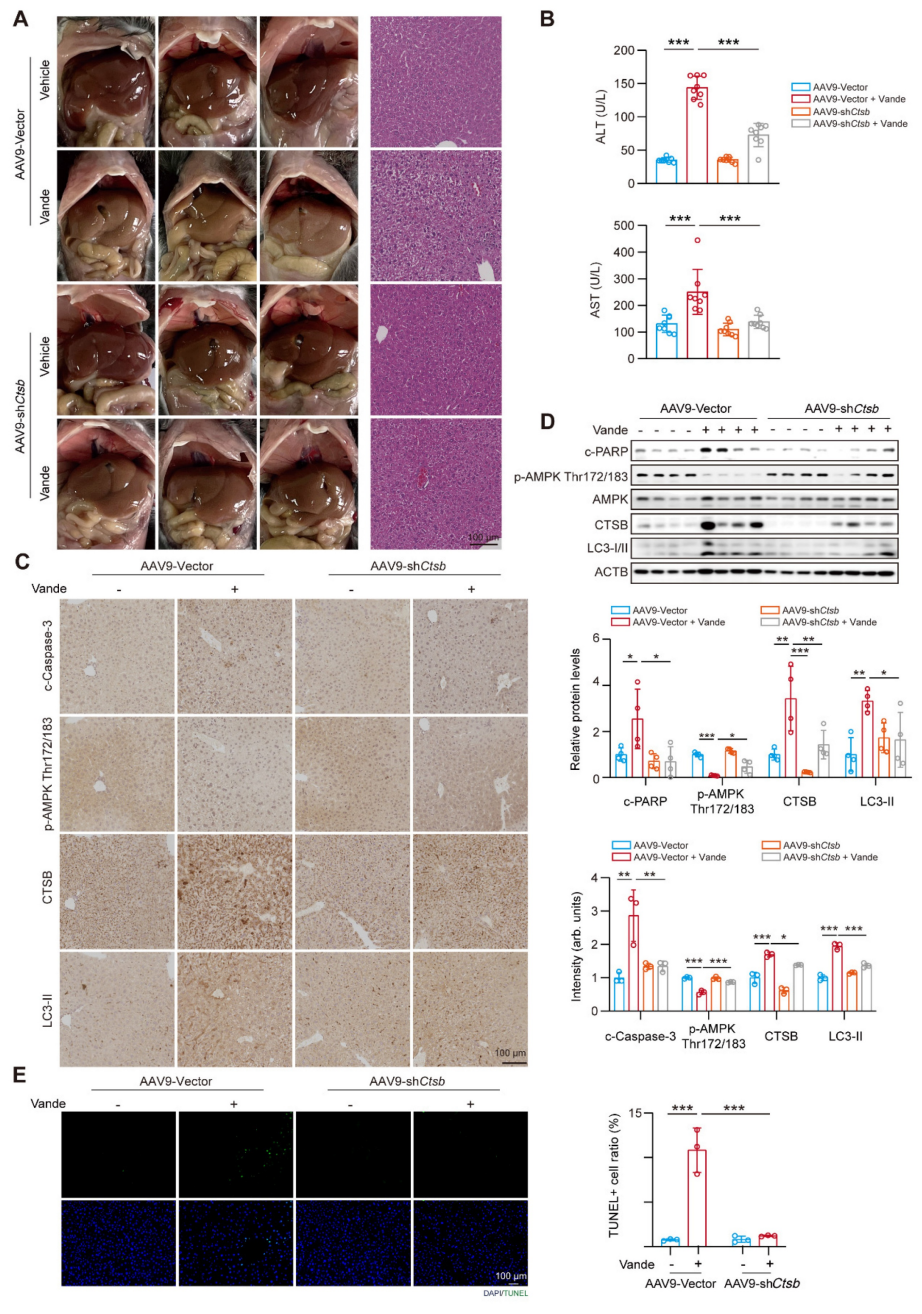
** Knockdown of CTSB ameliorated vandetanib-induced hepatotoxicity *in vivo.*** (**A-D**) C57BL/6 mice were randomly divided into 4 groups. After injection of AAV9-sh*Ctsb* adeno virus by tail vein for 3 weeks, mice were treated with 0.5% CMC-Na or 100 mg/kg/day vandetanib by gavage for another 4 weeks. (**A**) (Left panel) Representative photographs of mice liver. (Right panel) Representative images of H&E staining in liver tissues. Scale bar, 100 μm. (**B**) The levels of serum ALT and AST. (n = 8). (**C**) The expression levels of cleaved Caspase 3, p-AMPK Thr172/183, CTSB and LC3-II in liver tissues were detected by immunohistochemical analysis. Scale bar, 100 μm. (**D**) The expression levels of c-PARP, p-AMPK Thr172/183, AMPK, CTSB and LC3-I/II in liver tissues were measured by western blot. (n = 4). (**E**) Representative images of TUNEL staining in liver tissues. Scale bar, 100 μm. Data are represented as the mean ± SD. *p < 0.05, **p < 0.01, ***p < 0.001. One way ANOVA followed by Tukey post hoc test for (**B**), (**C**), (**D**) and (**E**).

To further investigate the role of CTSB in vandetanib-induced hepatotoxicity *in vivo*, we established a mouse model with liver *Ctsb* knockdown using an AAV9 adeno-associated virus carrying a broad-spectrum U6 promoter (referred to as AAV9-U6-sh*Ctsb*) known for its high hepatic and heart invasiveness. H&E staining revealed that cell shrinkage and tissue vacuolization in the mouse model with liver *Ctsb* knockdown were significantly mitigated (**Fig. 5A**). Meanwhile, the upregulation of ALT and AST levels induced by vandetanib was restored in mice injected with AAV9-U6-sh*Ctsb* virus (**Fig. 5B**). Next, to additionally elucidate the changes in downstream signaling pathways associated with vandetanib-induced liver injury upon *Ctsb* knockdown, we utilized immunohistochemistry and western blot to assess the alterations in cleaved Caspase 3, p-AMPK (Thr172/183), CTSB, and LC3-II protein expression. Compared to the vehicle group, the expression levels of cleaved Caspase 3, CTSB, and LC3-II were significantly upregulated in the liver tissues of mice in vandetanib treatment group, while p-AMPK (Thr172/183) levels were markedly downregulated. Mice injected with AAV9-U6-sh*Ctsb* and treated with vandetanib exhibited a notable restoration in the levels of these aberrant proteins (**Fig. 5C, D**). TUNEL staining demonstrated that CTSB knockdown rescued vandetanib-induced hepatocyte apoptosis (**Fig. 5E**). Taken together, the results collectively indicate that knockdown of CTSB ameliorates vandetanib-induced hepatotoxicity *in vivo*.

### inhibits CTSB activity to intervene in vandetanib-induced hepatotoxicity

**Figure 6:**
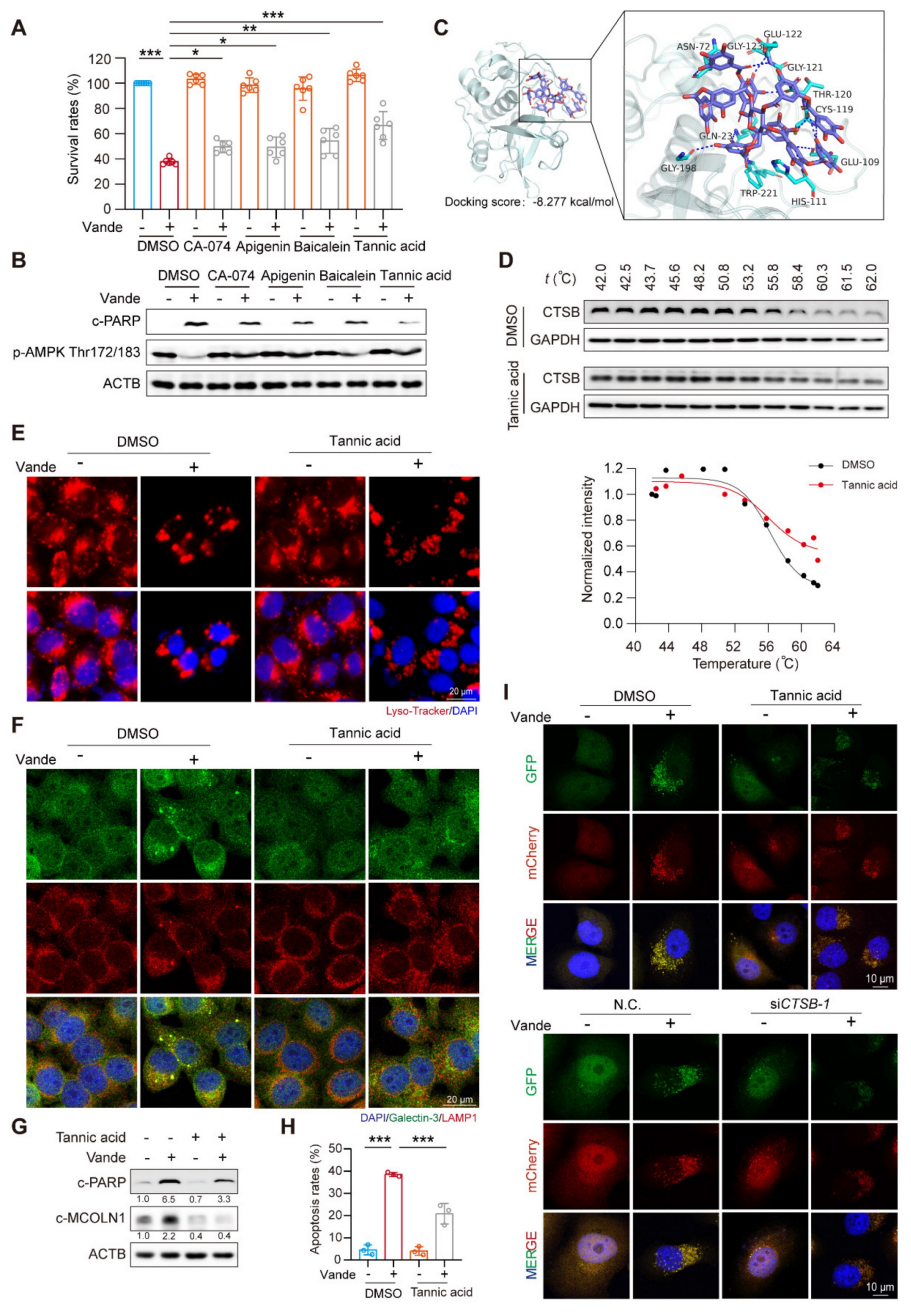
** Tannic acid inhibited vandetanib-induced hepatocyte death by direct binding to CTSB.** (**A-B**) HL-7702 cells were treated with 20 μM vandetanib and/or 5 μM CA-074, 5 μM Apigenin, 5 μM Baicalein, 5 μM Tannic acid for 36 h. (**A**) The survival rates of HL-7702 cells were measured by SRB staining. (n = 6). (**B**) The expression levels of c-PARP and p-AMPK Thr172/183 were analyzed by western blot. (**C**) Molecular docking of tannic acid and CTSB. (**D**) The binding stability determined by CETSA assay of drug molecules to proteins. (**E**) HL-7702 cells were treated with 20 μM vandetanib and/or 5 μM tannic acid for 36 h. Representative images of Lyso-Tracker staining in HL-7702 cells. Scale bar, 20 μm. (**F**) The expression levels of Galectin-3 and LAMP1 in HL-7702 cells treated with 20 μM vandetanib for 36 h were measured by immunofluorescence. Scale bar, 20 μm. (**G**) HL-7702 cells were treated with 20 μM vandetanib and/or 5 μM tannic acid for 36 h. The expression levels of c-PARP and c-MCOLN1 were measured by western blot. (**H**) HL-7702 cells were treated with 20 μM vandetanib and/or 5 μM tannic acid for 36 h. The apoptosis rates were detected by flow cytometry of Annexin V/PI staining. (n = 3). (**I**) Autophagic flux was assessed in HL-7702 cells transfected with Ad-mCherry-GFP-LC3B using confocal microscopy. (Upper) Cells were treated with 20 μM vandetanib and/or 5 μM tannic acid for 36 h. (Lower) Cells were transfected with negative control or CTSB-targeting siRNA followed by treatment with or without 20 μM vandetanib for 36 h. Scale bar, 10 μm. Data are represented as the mean ± SD. ***p < 0.001. One way ANOVA followed by Tukey post hoc test for (**A**) and (**H**).

Building on previous research that confirmed the efficacy of CA-074, apigenin, baicalein, and tannic acid in inhibiting CTSB activity 35, we focused on investigating the subsequent protective effects of these inhibitors against vandetanib-induced liver injury. We first evaluated the effects of these inhibitors on CTSB activity and the expression of its downstream target, c-MCOLN1. The results indicated that all inhibitors effectively suppressed CTSB activity and reduced c-MCOLN1 levels (**Supplementary Fig. 11A, B**). Concurrently, these CTSB inhibitors attenuated vandetanib-induced cell death and partially reversed the aberrant expression of c-PARP and p-AMPK (Thr172/183) caused by vandetanib, with tannic acid exhibiting the most pronounced effect (**Fig. 6A, B**). Furthermore, a dose-response assay demonstrated that tannic acid inhibited CTSB activity in a concentration-dependent manner and provided the most potent cytoprotection at a concentration of 5 μM (**Supplementary Fig. 11C, D**). Then we conducted molecular docking experiments to verify whether tannic acid functions through direct binding to CTSB. The overall binding score of tannic acid and CTSB was -8.277 kcal/mol, indicating a direct binding interaction between the two compounds. The hydrophobic interaction binding sites were identified as Asn-72 and Trp-221, while the hydrogen bonding interaction sites included His-111, Glu-122, Trp-221, Gly-121, Gly-123, Asn-72, Gly-198, Thr-120, Cys-119, Glu-109, and Gln-23 (**Fig. 6C**). Notably, His-111, Glu-122, Trp-221, and Gln-23 have been shown to be essential for the activity of CTSB 36-38. To further verify the interaction between tannic acid and CTSB, we employed cellular thermal shift assay (CETSA), a technique that facilitates studying drug candidate target engagement within a cellular context 39. Our results indicate that as the temperature increases, tannic acid markedly enhances the stability of CTSB, confirming that tannic acid inhibits CTSB activity through direct binding (**Fig. 6D**).

Based on these results, we selected tannic acid to investigate the protective effect of inhibiting CTSB activity against vandetanib-induced hepatotoxicity. We conducted a treatment of HL-7702 cells using vandetanib and/or tannic acid, subsequently assessing the functionality of lysosomes. The results indicated that the lysosomal swelling and aggregation induced by vandetanib alone were effectively alleviated by co-treatment with tannic acid (**Fig. 6E, F**). We then utilized western blot and flow cytometry to investigate the impact of tannic acid on vandetanib-induced apoptosis. The results demonstrated that tannic acid effectively alleviated vandetanib-induced apoptosis and restored the cleavage of MCOLN1 caused by excessive accumulation of CTSB (**Fig. 6G, H, Supplementary Fig. 12**). Furthermore, co-treatment with tannic acid alleviated the vandetanib-induced inhibition of autophagic flux, an effect comparable to that achieved by CTSB knockdown (**Fig. 6I**). This result indicated that tannic acid mitigated vandetanib-induced lysosomal dysfunction and autophagic flux blockade by inhibiting CTSB activity. Meanwhile, we measured p-STAT3 and p-JAK1 levels in mouse liver tissue and in cells treated with tannic acid, and found that tannic acid did not affect their expression levels **(Supplementary Fig. 13)**. Our findings suggested that the inhibitory effect of tannic acid acted by directly targeting CTSB activity downstream, independent of the upstream JAK/STAT3 signaling.

### Tannic acid ameliorates vandetanib induced hepatotoxicity *in vivo*

**Figure 7:**
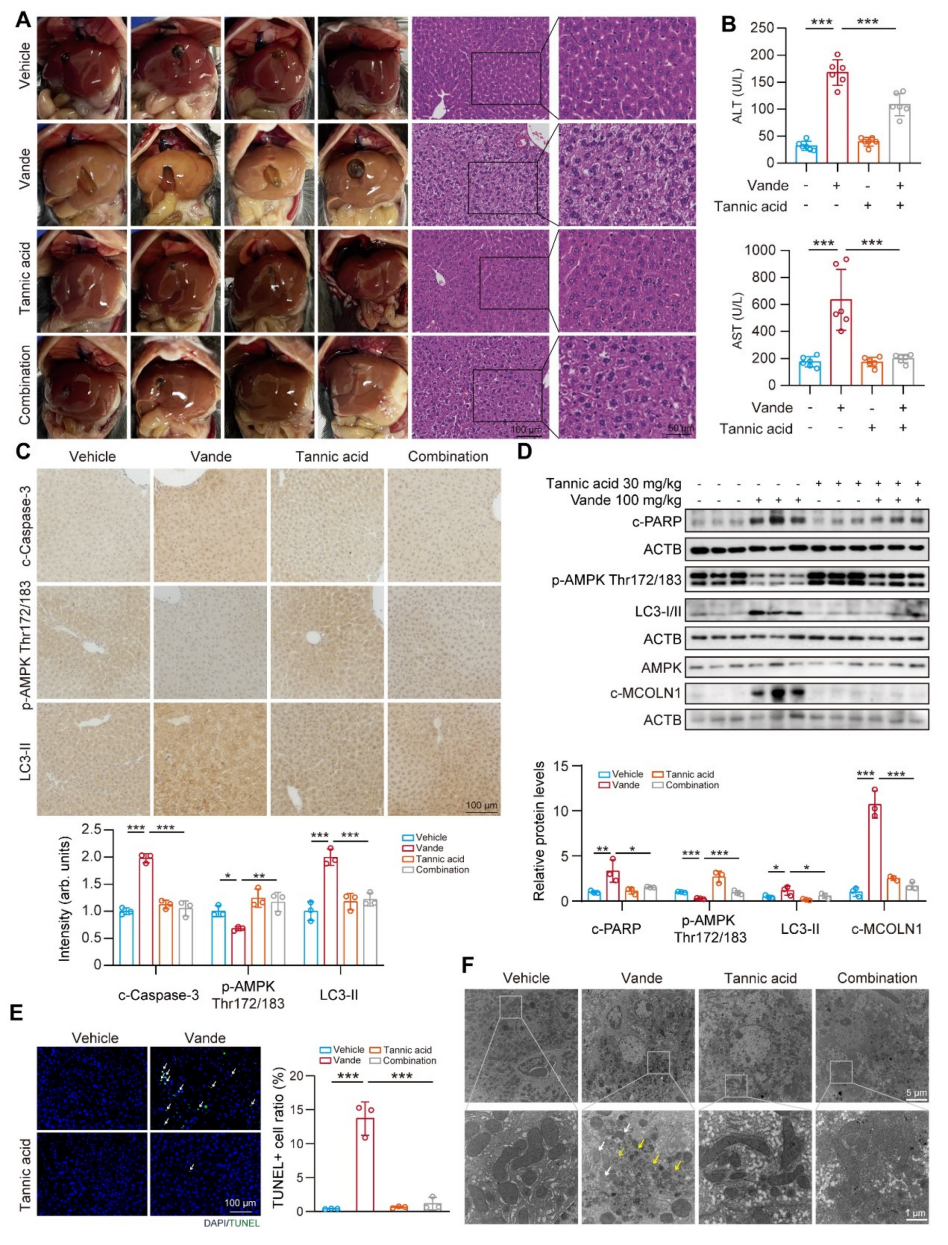
** Tannic acid ameliorated vandetanib induced hepatotoxicity *in vivo.*** (**A-E**) C57BL/6J mice were received 100 mg/kg vandetanib and/or 30 mg/kg tannic acid by gavage for 4 weeks. (**A**) (Left panel) Representative photographs of mice liver. (Right panel) Representative images of H&E staining in liver tissues. For 100× magnification, scale bar, 100 µm; for 200× magnification, scale bar, 50 μm. (**B**) The levels of serum ALT and AST were analyzed. (n = 6). (**C**) The expression levels of cleaved Caspase 3, p-AMPK Thr172/183 and LC3-II in liver tissues were detected by immunohistochemical analysis. Scale bar, 100 μm. (**D**) The expression levels of c-PARP, p-AMPK Thr172/183, LC3-I/II, AMPK and c-MOCLN1 were measured by western blot. (n = 3). (**E**) Representative images of TUNEL staining in liver tissues. Scale bar, 100 μm. (**F**) Transmission electron microscopy observation of the liver tissues. Representative images of mitochondrial structure were shown on the up (scale bar, 5 μm) and enlarged view was presented blow (scale bar, 1 μm). The yellow arrows: abnormal membrane structure organelles; the white arrows: damaged mitochondria. Data are represented as the mean ± SD. *p < 0.05, **p < 0.01, ***p < 0.001. One way ANOVA followed by Tukey post hoc test for (**B**), (**C**), (**D**) and (**E**).

Next, we aimed to examine the protective role of tannic acid *in vivo*. C57BL/6J mice were administered tannic acid at a dosage of 30 mg/kg, either alone or in combination with 100 mg/kg of vandetanib, over a period of 4 weeks. At the conclusion of the treatment period, serum and liver samples were collected for analysis. Histopathological evaluation via H&E staining demonstrated tannic acid-mediated preservation of hepatic cytoarchitecture, with significant attenuation of vandetanib-induced histoarchitectural perturbations (**Fig. 7A**). In addition, serum levels of ALT and AST were significantly restored in mice treated with a combination treatment of tannic acid and vandetanib, in comparison to those treated solely with vandetanib (**Fig. 7B**). Co-treatment of tannic acid and vandetanib restored protein levels of cleaved Caspase 3, c-PARP, p-AMPK (Thr172/183), cleaved-MCOLN1, and LC3-II compared to vandetanib alone (**Fig. 7C, D**). TUNEL staining demonstrated that tannic acid co-treatment significantly attenuated vandetanib-induced hepatocyte apoptosis (**Fig. 7E**). To evaluate the mitochondrial protective capacity of tannic acid, ultrastructural analysis via transmission electron microscopy (TEM) was performed. Hepatocytes from vandetanib-treated cohorts exhibited pronounced mitochondrial pathology characterized by proliferation of multilamellar bodies and markedly shortened mitochondria with structural disarray. In contrast, TA coadministration substantially attenuated these ultrastructural pathologies (**Fig. 7F**). These findings conclusively establish the mitochondrial protective efficacy of TA against vandetanib-induced hepatotoxicity.

### Targeting CTSB also attenuates vandetanib-induced cardiotoxicity

**Figure 8:**
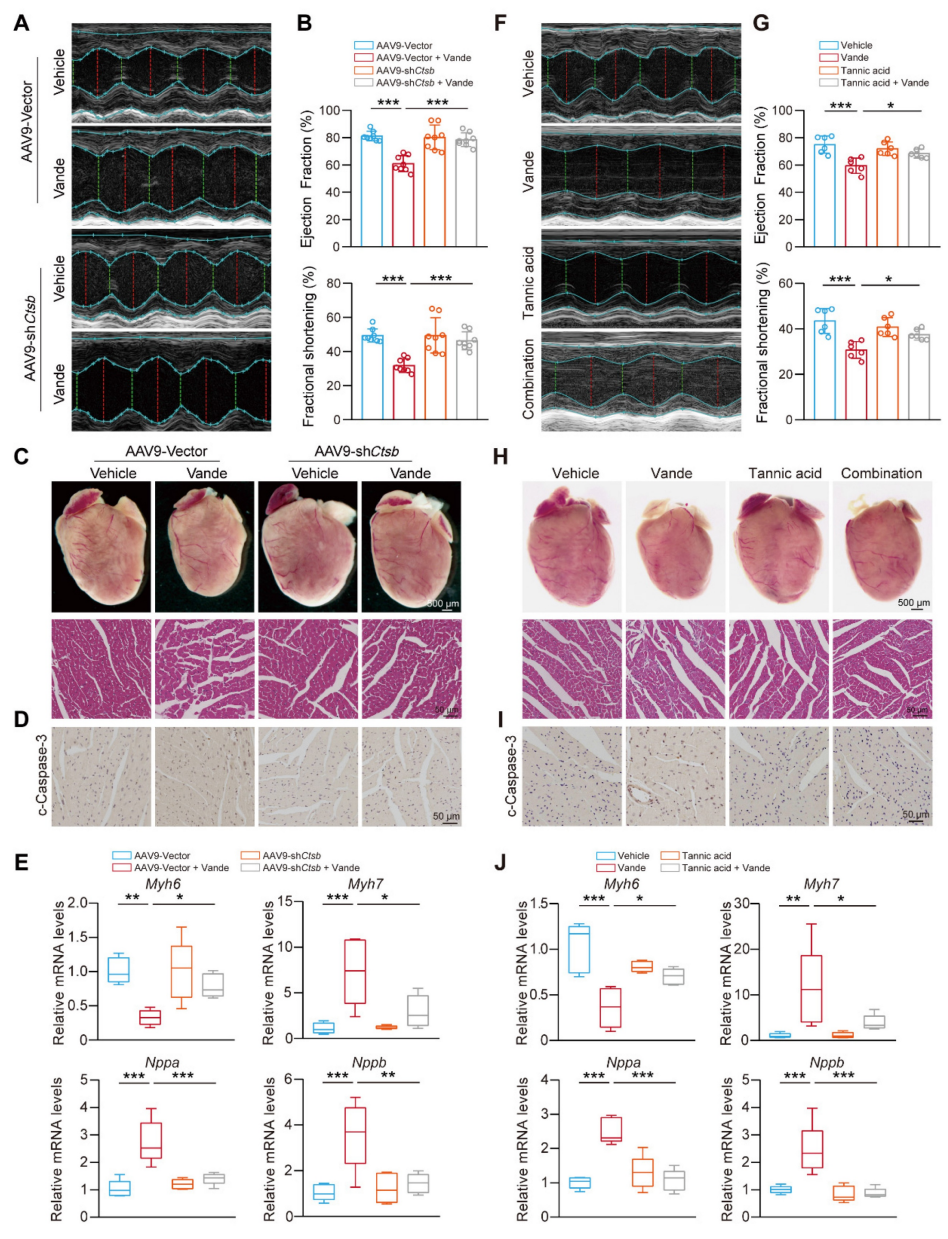
** Targeting CTSB alleviated vandetanib-induced cardiac injury.** (**A-E**) C57BL/6 mice were randomly divided into 4 groups (n = 8 per group). After injection of AAV9-sh*Ctsb* adeno virus by tail vein for 3 weeks, mice were treated with 0.5% CMC-Na or 100 mg/kg/day vandetanib by gavage for another 4 weeks. (**A**) Representative M-mode echocardiogram images. (**B**) Quantifications of Ejection fraction and Fractional shortening ratios. (**C**) (Upper photos) Representative photographs of heart tissues. Scale bar, 500 μm. (Lower photos) H&E staining of heart tissues. Scale bar, 50 μm. (**D**) Representative images of immunohistochemistry for cleaved Caspase 3 staining in heart tissues. Scale bar: 50 μm. (**E**) Total RNA was extracted from mice hearts and the expression levels of *Myh6*, *Myh7*, *Nppa* and *Nppb* were measured by qRT-PCR. (**F-J**) C57BL/6 mice were randomly divided into 4 groups (n = 6 per group). The C57BL/6J mice were received 100 mg/kg vandetanib and/or 30 mg/kg tannic acid for 4 weeks. (**F**) Representative M-mode echocardiogram images. (**G**) Quantifications of Ejection fraction and Fractional shortening ratios. (**H**) (Upper photos) Representative photographs of heart tissues. Scale bar, 500 μm. (Lower photos) H&E staining of heart tissues. Scale bar, 50 μm. (**I**) Representative images of immunohistochemistry for cleaved Caspase 3 staining in heart tissues. Scale bar: 50 μm. (**J**) Total RNA was extracted from mice hearts and the expression levels of *Myh6*, *Myh7*, *Nppa* and *Nppb* were measured by qRT-PCR. Data are represented as the mean ± SD. *p < 0.05, **p < 0.01, ***p < 0.001. One way ANOVA followed by Tukey post hoc test for (**B**), (**E**), (**G**) and (**J**).

Building on the *in vivo* animal models previously established, we focused on the cardiac effects of *Ctsb* knockdown. Cardiac function evaluation revealed that *Ctsb* knockdown mice showed significant attenuation of vandetanib-induced cardiac dysfunction, as evidenced by preserved LVEF and LVFS ratios (**Fig. 8A, B**). Morphologically, *Ctsb* knockdown reversed vandetanib-induced cardiac atrophy and ameliorated pathological changes in myocardial tissue (**Fig. 8C**). Consistent with these functional and morphological improvements, *Ctsb* knockdown suppressed the activation of cleaved Caspase 3 (**Fig. 8D**) and normalized the expression of cardiac remodeling-related genes (**Fig. 8E**).

We next examined whether tannic acid could recapitulate the cardioprotective effects observed with genetic *Ctsb* knockdown. Strikingly, co-administration of tannic acid with vandetanib mirrored the effects of *Ctsb* knockdown, significantly preserving LVEF and LVFS ratios (**Fig. 8F, G**), mitigating cardiac atrophy, and reducing histopathological damage (**Fig. 8H**). Tannic acid similarly attenuated cleaved Caspase 3 upregulation (**Fig. 8I**) and restored normal expression of cardiac remodeling genes (**Fig. 8J**).

Overall, these data establish its critical role in vandetanib-associated cardiotoxicity. We demonstrate that genetic knockdown of *Ctsb* or pharmacological inhibition with tannic acid effectively attenuates cardiomyocyte apoptosis, thereby preserving cardiac function and mitigating pathological remodeling. These results unify CTSB as a central driver of both hepatic and cardiac toxicity induced by vandetanib. Importantly, the concordant cardioprotective effects of tannic acid and *Ctsb* knockdown underscore the therapeutic viability of targeting CTSB-dependent apoptosis to counteract vandetanib's life-threatening complications.

## Discussion

The hepato-cardiotoxicity of antitumor agents have persisted as pressing toxicological challenges that restrict their clinical application. Elucidating the critical link between these dual-organ toxicities through research will not only overcome current clinical toxicity limitations but also furnish strategic insights for developing next-generation therapeutic agents. In this study, we revealed that vandetanib-induced hepatocardiotoxicity are associated with mitochondria-dependent apoptosis. Further results elucidate that the observed cell death results from the accumulation of CTSB induced by STAT3 activation, which subsequently leads to lysosomal dysfunction and autophagy inhibition. Unexpectedly, inhibiting the initiation of autophagy only partially alleviate vandetanib-induced apoptosis. Instead, we turn to CTSB and identify one of its inhibitors, tannic acid, has a good protective effect against the liver damage by directly binding to CTSB, demonstrating CTSB as a key factor and tannic acid as a potential strategy for vandetanib-induced hepatocadiotoxicity.

Accumulating evidence establishes CTSB as a multifunctional protease implicated in diverse pathophysiological processes spanning oncogenesis 40, autoimmune and renal diseases, joint disorders, osteoporosis, cardiovascular diseases 41, neurodegenerative diseases 42, and obesity 43. Recent studies have shown that exercise modulates CTSB, thereby improving spatial memory in rodents and humans 17. CTSB catalyzes C-terminal truncation of epidermal and insulin-like growth factor, negatively regulating their signaling pathways. Additionally, CTSB plays a neuroprotective role by inhibiting amyloidogenesis through C-terminal protein truncation of Aβ 44. When CTSB activity is inhibited, it directly damages lysosomal degradation function. Conversely, excessive or mislocalized CTSB activity can destabilize lysosomal membranes and promote lysosomal membrane permeabilization, leading to the release of cathepsins into the cytosol and amplification of mitochondrial outer membrane permeabilization-driven apoptotic signaling 45. The role of lysosomal dysfunction in human diseases goes beyond rare inherited diseases, such as lysosomal storage disorders, to include common neurodegenerative and metabolic diseases, as well as cancer. Genetic and clinical studies further indicate that even partial impairments in lysosomal acidification, hydrolase activity or membrane trafficking are sufficient to drive progressive neurodegeneration and systemic pathology 46. In Alzheimer disease, mutations in presenilin one result in loss of acidification, impairing TRPML1 activity and leading to endo-lysosome accumulation in dystrophic axons 47. Hepatic lipid metabolism studies reveal that cholesterol overload disrupts lysosomal SNARE complex functionality, inducing autophagic flux impairment, a hallmark of progressive NAFLD 48. Lysosomal destruction inhibits the uptake of low molecular weight proteins and nutrients by renal proximal tubular epithelial cells and leads to chronic kidney disease 49. Our findings delineate a novel toxicological mechanism wherein vandetanib-induced STAT3 phosphorylation at Tyr705 drives CTSB transcriptional activation, precipitating apoptotic cascades via CTSB overexpression-mediated lysosomal dysfunction. Despite STAT3's pleiotropic physiological functions, clinical translation of STAT3 inhibitors remains constrained by on-target toxicity concerns. Given the compelling mechanistic linkage between STAT3 signaling and CTSB transcriptional regulation, the adverse effect profiles of STAT3 inhibitors warrant reassessment, as their toxicity may stem from lysosomal dysfunction.

Calcium homeostasis is critical for lysosomal function, particularly membrane trafficking 50. As with the synaptic vesicle fusion with the plasma membrane, lysosome fusion with other membranes is specifically Ca^2+^ dependent. For example, the fast Ca^2+^ chelator BAPTA inhibits the fusion of lysosomes with other organelles 51, 52. Dysregulation of lysosomal Ca^2+^ release leads to the accumulation of damaged macromolecules, impaired organelles, and intracellular storage 53. Three main types of Ca^2+^ channels have been identified in the lysosomal membrane of mammalian cells: transient receptor potential cation channels of the mucolipin family (TRPML), two-pore channels (TPC), and the trimeric Ca^2+^ two-transmembrane channel P2X4^54^, ^55^. Our data demonstrated significant activation of lysosomal pathways and calcium signaling following vandetanib exposure, a phenomenon mediated through CTSB-dependent proteolytic processing of MCOLN1, a TRP superfamily cation channel critical for lysosomal calcium homeostasis. This mechanism may underlie the observed ultrastructural pathology of membranous organelle disorganization observed in vandetanib-treated hepatocytes. Meanwhile, given calcium homeostasis' critical role in cardiac contractility, CTSB-mediated MCOLN1 over-cleavage may underlie vandetanib-induced QT interval prolongation and Torsades de pointes, warranting dedicated investigation into the mechanistic role of CTSB in these pathologies. Mutations in the human MCOLN1 gene result in mucolipidosis type IV (ML4). Cells from ML4 patients have defective membrane trafficking in the late endocytosis pathway, lysosomal Ca^2+^ overload 56, enlarged lysosomes 57, impaired lysosome biogenesis, and defective apoptotic cell clearance 58. Given the membrane-regulatory role of lysosomal Ca^2+^, the inhibition of autophagy caused by vandetanib may be also particularly attributed to the excessive cleavage of MCOLN1. Further investigation is warranted to elucidate the precise molecular mechanisms, particularly the involvement of key regulators such as SNARE complexes, STX17, VAMP8, and SNAP29^59^, ^60^, ​​in vandetanib-induced blockade of autophagosome-lysosome fusion.

Studies have shown that AMPK is located in lysosomes through the interaction with the axin protein and can be directly phosphorylated on Thr172/183 in response to calcium flux by the calcium-sensitive kinase CAMKK2 (also known as CAMKKβ)^61^. Thus, calcium signaling is linked to the regulation of energy metabolism by AMPK 62. AMPK activation is essential for regulating mitochondrial dynamics and regulates mitochondria-dependent apoptosis. Studies have reported that AMPK can phosphorylate Ser155 and Ser173, two phosphorylation sites of MEF, which regulates mitochondrial contraction and is a central component of the mitochondrial fission process. Inhibition of AMPK signaling results in the impairment of mitochondrial fission and delays in the clearance of damaged mitochondria, ultimately promoting mitochondria-dependent apoptosis 63, 64. In our study, vandetanib triggered mitochondrial swelling and ultrastructural abnormalities, and restoration of AMPK phosphorylation at residues 172/183—achieved via AMPK-S183D plasmid overexpression or co-treatment with tannic acid to inhibit CTSB proteolytic activity—reversed these morphological aberrations and restored mitochondrial integrity. Co-treatment with CTSB inhibitors partially restores phosphorylation of AMPK at Thr172/183, further confirming AMPK as a downstream effector of CTSB signaling. The identification of this CTSB-AMPK regulatory axis demonstrates that targeted CTSB inhibition enables precise modulation of AMPK pathway activity, providing a novel therapeutic strategy to mitigate drug-induced hepatotoxicity and cardiotoxicity.

Our findings suggest that targeting CTSB inhibition may serve as a potential therapeutic strategy for vandetanib-induced hepatotoxicity and cardiotoxicity. Through systematic screening, we identified that tannic acid, a reported CTSB inhibitor, possesses protective properties against the toxic effects of vandetanib. In recent decades, extensive evidence has demonstrated that tannic acid can protect against the development of chronic diseases, such as cancer, cardiovascular disease, and neurodegeneration, by regulating inflammation and oxidative stress 65, 66. Mechanistically, tannic acid might activate the Keap1-Nrf2/ARE pathway to alleviate arsenic trioxide-induced liver injury 67 and increase endothelial nitric oxide synthase and nitric oxide levels to protect mice from carbon tetrachloride-induced liver damage 68. In neurodegeneration diseases, tannic acid was reported as an important lead compound against AD, while underlying mechanisms remain unknown. Based on the result that low levels of CTSB partially mitigate the progression of AD, it can be inferred that tannic acid might exert its neuroprotective effect by inhibiting CTSB. Our data that tannic acid directly interacts with CTSB through its enzymatic activity-related sites further confirms this hypothesis. Beyond the previously reported CTSB active sites (His-111, Glu-122, Trp-221, Gln-23), tannic acid exhibits additional binding interactions with residues Asn-72, Gly-121, Gly-123, Gly-198, Thr-120, Cys-119, and Glu-109. Systematic characterization of these sites will refine our understanding of CTSB catalytic determinants and accelerate clinical-grade inhibitor development.

Our study reveals for the first time that vandetanib-induced CTSB accumulation and lysosomal damage contribute to liver and cardiac damage *in vitro* and *in vivo*. We further found that up-regulated CTSB disrupts lysosomal calcium ion flow through cleavage of MCOLN1. This disruption inhibits the phosphorylation of AMPK Thr172/183, thereby triggering mitochondria-dependent apoptosis. Additionally, tannic acid, capable of inhibiting CTSB activity, may serve as a safe and effective strategy for cancer treatment.

## Supplementary Material

Supplementary figures and tables.

## References

[1] Wu J, Fan D, Shao Z, Xu B, Ren G, Jiang Z (2022). CACA Guidelines for Holistic Integrative Management of Breast Cancer. Holist Integr Oncol.

[2] Yoh K, Seto T, Satouchi M, Nishio M, Yamamoto N, Murakami H (2017). Vandetanib in patients with previously treated RET-rearranged advanced non-small-cell lung cancer (LURET): an open-label, multicentre phase 2 trial. Lancet Respir Med.

[3] Scheffel RS, Dora JM, Siqueira DR, Burttet LM, Cerski MR, Maia AL (2013). Toxic cardiomyopathy leading to fatal acute cardiac failure related to vandetanib: a case report with histopathological analysis. Eur J Endocrinol.

[4] Hou W, Ding M, Li X, Zhou X, Zhu Q, Varela-Ramirez A (2021). Comparative evaluation of cardiovascular risks among nine FDA-approved VEGFR-TKIs in patients with solid tumors: a Bayesian network analysis of randomized controlled trials. J Cancer Res Clin Oncol.

[5] Shah RR, Morganroth J, Shah DR (2013). Hepatotoxicity of tyrosine kinase inhibitors: clinical and regulatory perspectives. Drug Saf.

[6] Weil A, Martin P, Smith R, Oliver S, Langmuir P, Read J (2010). Pharmacokinetics of vandetanib in subjects with renal or hepatic impairment. Clin Pharmacokinet.

[7] Zhang Z, Yue P, Lu T, Wang Y, Wei Y, Wei X (2021). Role of lysosomes in physiological activities, diseases, and therapy. J Hematol Oncol.

[8] Gukovskaya AS, Gukovsky I, Algul H, Habtezion A (2017). Autophagy, Inflammation, and Immune Dysfunction in the Pathogenesis of Pancreatitis. Gastroenterology.

[9] Zhai Y, Chen L, Zhao Q, Zheng ZH, Chen ZN, Bian H (2023). Cysteine carboxyethylation generates neoantigens to induce HLA-restricted autoimmunity. Science.

[10] Gros F, Muller S (2023). The role of lysosomes in metabolic and autoimmune diseases. Nat Rev Nephrol.

[11] Wilson JS, Apte MV, Thomas MC, Haber PS, Pirola RC (1992). Effects of ethanol, acetaldehyde and cholesteryl esters on pancreatic lysosomes. Gut.

[12] Bremer C, Tung CH, Bogdanov A Jr, Weissleder R (2002). Imaging of differential protease expression in breast cancers for detection of aggressive tumor phenotypes. Radiology.

[13] Rozman J, Stojan J, Kuhelj R, Turk V, Turk B (1999). Autocatalytic processing of recombinant human procathepsin B is a bimolecular process. FEBS Lett.

[14] Werneburg NW, Guicciardi ME, Bronk SF, Kaufmann SH, Gores GJ (2007). Tumor necrosis factor-related apoptosis-inducing ligand activates a lysosomal pathway of apoptosis that is regulated by Bcl-2 proteins. J Biol Chem.

[15] Wuopio J, Hilden J, Bring C, Kastrup J, Sajadieh A, Jensen GB (2018). Cathepsin B and S as markers for cardiovascular risk and all-cause mortality in patients with stable coronary heart disease during 10 years: a CLARICOR trial sub-study. Atherosclerosis.

[16] Dai J, Zhang Q, Wan C, Liu J, Zhang Q, Yu Y (2021). Significances of viable synergistic autophagy-associated cathepsin B and cathepsin D (CTSB/CTSD) as potential biomarkers for sudden cardiac death. BMC Cardiovasc Disord.

[17] Moon HY, Becke A, Berron D, Becker B, Sah N, Benoni G (2016). Running-Induced Systemic Cathepsin B Secretion Is Associated with Memory Function. Cell Metab.

[18] Mazo CE, Miranda ER, Shadiow J, Vesia M, Haus JM (2022). High Intensity Acute Aerobic Exercise Elicits Alterations in Circulating and Skeletal Muscle Tissue Expression of Neuroprotective Exerkines. Brain Plast.

[19] Wu Y, Mumford P, Noy S, Cleverley K, Mrzyglod A, Luo D (2023). Cathepsin B abundance, activity and microglial localisation in Alzheimer's disease-Down syndrome and early onset Alzheimer's disease; the role of elevated cystatin B. Acta Neuropathol Commun.

[20] Yuyama K, Sun H, Fujii R, Hemmi I, Ueda K, Igeta Y (2024). Extracellular vesicle proteome unveils cathepsin B connection to Alzheimer's disease pathogenesis. Brain.

[21] Czabotar PE, Garcia-Saez AJ (2023). Mechanisms of BCL-2 family proteins in mitochondrial apoptosis. Nat Rev Mol Cell Biol.

[22] Xiong S, Mu T, Wang G, Jiang X (2014). Mitochondria-mediated apoptosis in mammals. Protein Cell.

[23] Fernandez-Patron C, Lopaschuk GD, Hardy E (2024). A self-reinforcing cycle hypothesis in heart failure pathogenesis. Nat Cardiovasc Res.

[24] Fang W, Deng Z, Benadjaoud F, Yang C, Shi GP (2020). Cathepsin B deficiency ameliorates liver lipid deposition, inflammatory cell infiltration, and fibrosis after diet-induced nonalcoholic steatohepatitis. Transl Res.

[25] Cui Z, Zeng C, Huang F, Yuan F, Yan J, Zhao Y (2022). Cas13d knockdown of lung protease Ctsl prevents and treats SARS-CoV-2 infection. Nat Chem Biol.

[26] Zhao J, Qi YF, Yu YR (2021). STAT3: A key regulator in liver fibrosis. Ann Hepatol.

[27] Martinez-Fabregas J, Prescott A, van Kasteren S, Pedrioli DL, McLean I, Moles A (2018). Lysosomal protease deficiency or substrate overload induces an oxidative-stress mediated STAT3-dependent pathway of lysosomal homeostasis. Nat Commun.

[28] Ruiz-Blazquez P, Pistorio V, Fernandez-Fernandez M, Moles A (2021). The multifaceted role of cathepsins in liver disease. J Hepatol.

[29] Rafn B, Nielsen CF, Andersen SH, Szyniarowski P, Corcelle-Termeau E, Valo E (2012). ErbB2-driven breast cancer cell invasion depends on a complex signaling network activating myeloid zinc finger-1-dependent cathepsin B expression. Mol Cell.

[30] Park S, Scheffler TL, Rossie SS, Gerrard DE (2013). AMPK activity is regulated by calcium-mediated protein phosphatase 2A activity. Cell Calcium.

[31] Xu Z, Pan Z, Jin Y, Gao Z, Jiang F, Fu H (2024). Inhibition of PRKAA/AMPK (Ser485/491) phosphorylation by crizotinib induces cardiotoxicity via perturbing autophagosome-lysosome fusion. Autophagy.

[32] Luo P, Yan H, Du J, Chen X, Shao J, Zhang Y (2021). PLK1 (polo like kinase 1)-dependent autophagy facilitates gefitinib-induced hepatotoxicity by degrading COX6A1 (cytochrome c oxidase subunit 6A1). Autophagy.

[33] Xu Z, Jin Y, Gao Z, Zeng Y, Du J, Yan H (2022). Autophagic degradation of CCN2 (cellular communication network factor 2) causes cardiotoxicity of sunitinib. Autophagy.

[34] Li J, Yin K, Hou L, Zhang Y, Lu H, Ma C (2023). Polystyrene microplastics mediate inflammatory responses in the chicken thymus by Nrf2/NF-kappaB pathway and trigger autophagy and apoptosis. Environ Toxicol Pharmacol.

[35] Gu S, Tan J, Li Q, Liu S, Ma J, Zheng Y (2020). Downregulation of LAPTM4B Contributes to the Impairment of the Autophagic Flux via Unopposed Activation of mTORC1 Signaling During Myocardial Ischemia/Reperfusion Injury. Circ Res.

[36] Chitranshi N, Kumar A, Sheriff S, Gupta V, Godinez A, Saks D (2021). Identification of Novel Cathepsin B Inhibitors with Implications in Alzheimer's Disease: Computational Refining and Biochemical Evaluation. Cells.

[37] Pan X, Tan N, Zeng G, Zhang Y, Jia R (2005). Amentoflavone and its derivatives as novel natural inhibitors of human Cathepsin B. Bioorg Med Chem.

[38] Portaro FC, Santos AB, Cezari MH, Juliano MA, Juliano L, Carmona E (2000). Probing the specificity of cysteine proteinases at subsites remote from the active site: analysis of P4, P3, P2' and P3' variations in extended substrates. Biochem J.

[39] Jafari R, Almqvist H, Axelsson H, Ignatushchenko M, Lundback T, Nordlund P (2014). The cellular thermal shift assay for evaluating drug target interactions in cells. Nat Protoc.

[40] Bruchard M, Mignot G, Derangere V, Chalmin F, Chevriaux A, Vegran F (2013). Chemotherapy-triggered cathepsin B release in myeloid-derived suppressor cells activates the Nlrp3 inflammasome and promotes tumor growth. Nat Med.

[41] Liu CL, Guo J, Zhang X, Sukhova GK, Libby P, Shi GP (2018). Cysteine protease cathepsins in cardiovascular disease: from basic research to clinical trials. Nat Rev Cardiol.

[42] Kim KR, Cho EJ, Eom JW, Oh SS, Nakamura T, Oh CK (2022). S-Nitrosylation of cathepsin B affects autophagic flux and accumulation of protein aggregates in neurodegenerative disorders. Cell Death Differ.

[43] Araujo TF, Cordeiro AV, Vasconcelos DAA, Vitzel KF, Silva VRR (2018). The role of cathepsin B in autophagy during obesity: A systematic review. Life Sci.

[44] Oberstein TJ, Utz J, Spitzer P, Klafki HW, Wiltfang J, Lewczuk P (2020). The Role of Cathepsin B in the Degradation of Abeta and in the Production of Abeta Peptides Starting with Ala2 in Cultured Astrocytes. Front Mol Neurosci.

[45] Wang F, Gomez-Sintes R, Boya P (2018). Lysosomal membrane permeabilization and cell death. Traffic.

[46] Gegg ME, Schapira AHV (2022). Lysosomal-endolysomal dysfunction and neurodegenerative diseases. Neurobiol Dis.

[47] Settembre C, Perera RM (2024). Lysosomes as coordinators of cellular catabolism, metabolic signalling and organ physiology. Nat Rev Mol Cell Biol.

[48] Fraldi A, Annunziata F, Lombardi A, Kaiser HJ, Luis Medina D, Spampanato C (2022). Lysosomal fusion and SNARE function are impaired by cholesterol accumulation in lysosomal storage disorders. EMBO J.

[49] Festa BP, Berquez M, Nieri D, Luciani A (2023). Endolysosomal Disorders Affecting the Proximal Tubule of the Kidney: New Mechanistic Insights and Therapeutics. Rev Physiol Biochem Pharmacol.

[50] Luzio JP, Pryor PR, Bright NA (2007). Lysosomes: fusion and function. Nat Rev Mol Cell Biol.

[51] Peters C, Mayer A (1998). Ca2+/calmodulin signals the completion of docking and triggers a late step of vacuole fusion. Nature.

[52] Lloyd-Evans E, Platt FM (2011). Lysosomal Ca(2+) homeostasis: role in pathogenesis of lysosomal storage diseases. Cell Calcium.

[53] Shen D, Wang X, Li X, Zhang X, Yao Z, Dibble S (2012). Lipid storage disorders block lysosomal trafficking by inhibiting a TRP channel and lysosomal calcium release. Nat Commun.

[54] Morgan AJ, Platt FM, Lloyd-Evans E, Galione A (2011). Molecular mechanisms of endolysosomal Ca2+ signalling in health and disease. Biochem J.

[55] Li P, Gu M, Xu H (2019). Lysosomal Ion Channels as Decoders of Cellular Signals. Trends Biochem Sci.

[56] Cao Q, Zhong XZ, Zou Y, Zhang Z, Toro L, Dong XP (2015). BK Channels Alleviate Lysosomal Storage Diseases by Providing Positive Feedback Regulation of Lysosomal Ca2+ Release. Dev Cell.

[57] Chen CS, Bach G, Pagano RE (1998). Abnormal transport along the lysosomal pathway in mucolipidosis, type IV disease. Proc Natl Acad Sci U S A.

[58] Zhong XZ, Yang Y, Sun X, Dong XP (2017). Methods for monitoring Ca(2+) and ion channels in the lysosome. Cell Calcium.

[59] Jian F, Wang S, Tian R, Wang Y, Li C, Li Y (2024). The STX17-SNAP47-VAMP7/VAMP8 complex is the default SNARE complex mediating autophagosome-lysosome fusion. Cell Res.

[60] Wang J, Su Q, Chen K, Wu Q, Ren J, Tang W (2024). Pyrimethamine upregulates BNIP3 to interfere SNARE-mediated autophagosome-lysosomal fusion in hepatocellular carcinoma. J Pharm Anal.

[61] Zong Y, Zhang CS, Li M, Wang W, Wang Z, Hawley SA (2019). Hierarchical activation of compartmentalized pools of AMPK depends on severity of nutrient or energy stress. Cell Res.

[62] Marcelo KL, Means AR, York B (2016). The Ca(2+)/Calmodulin/CaMKK2 Axis: Nature's Metabolic CaMshaft. Trends Endocrinol Metab.

[63] Shirihai OS, Song M, Dorn GW 2nd (2015). How mitochondrial dynamism orchestrates mitophagy. Circ Res.

[64] Pryde KR, Smith HL, Chau KY, Schapira AH (2016). PINK1 disables the anti-fission machinery to segregate damaged mitochondria for mitophagy. J Cell Biol.

[65] Yeung YT, Aziz F, Guerrero-Castilla A, Arguelles S (2018). Signaling Pathways in Inflammation and Anti-inflammatory Therapies. Curr Pharm Des.

[66] Tikoo K, Sane MS, Gupta C (2011). Tannic acid ameliorates doxorubicin-induced cardiotoxicity and potentiates its anti-cancer activity: potential role of tannins in cancer chemotherapy. Toxicol Appl Pharmacol.

[67] Jin W, Xue Y, Xue Y, Han X, Song Q, Zhang J (2020). Tannic acid ameliorates arsenic trioxide-induced nephrotoxicity, contribution of NF-kappaB and Nrf2 pathways. Biomed Pharmacother.

[68] Li M, Liu P, Xue Y, Liang Y, Shi J, Han X (2020). Tannic acid attenuates hepatic oxidative stress, apoptosis and inflammation by activating the Keap1-Nrf2/ARE signaling pathway in arsenic trioxide-toxicated rats. Oncol Rep.

