# Supplementary material for: Inhibition of Cathepsin B protects against vandetanib-induced hepato-cardiotoxicity by restoring lysosomal damage
Source: Int J Biol Sci. 2026 Jan 15;22(4):1752–74. doi: 10.7150/ijbs.122904 (PMC12905580; doi:10.7150/ijbs.122904)
Supplement: Supplementary file 1 — Supplementary figures and tables. [file ijbsv22p1752s1.pdf]

Inhibition of Cathepsin B protects against vandetanib-induced hepato-cardiotoxicity by restoring lysosomal damage

Wentong Wu, PhD [1,2,#], Jiangxia Du, PhD [3,#], Jinjin Li, BS [4], Shaoyin Zhang, MM [4], Xingchen Kang, BS [4], Yashi Cao, BS [4], Jian Chen, BS [4], Zengyue Pan, BS [4], Xiangliang Huang, BS [4], Zhifei Xu, PhD [4], Bo Yang, PhD [5,6], Qiaojun He, PhD [2,4,6], Xiaochun Yang, PhD [2,4,7,8], Hao Yan, PhD [4,*], Peihua Luo, PhD [1,2,4,*]

[1]Department of Cardiology, The Second Affiliated Hospital, Zhejiang University School of Medicine, Hangzhou, Zhejiang 310009, China

[2]Innovation Institute for Artificial Intelligence in Medicine of Zhejiang University, Hangzhou 310018, China

[3]Innovation in Digestive System Tumors, Ministry of Education, The Second Affiliated Hospital, Zhejiang University School of Medicine, Hangzhou 310017, China

[4]Center for Drug Safety Evaluation and Research of Zhejiang University, College of Pharmaceutical Sciences, Zhejiang University, Hangzhou 310058, China

[5]Institute of Pharmacology & Toxicology, College of Pharmaceutical Sciences, Zhejiang University, Hangzhou 310058, China

[6]School of Medicine, Hangzhou City University, Hangzhou 310015, China

[7]Nanhu Brain-computer Interface Institute, Hangzhou 311100, China

[8]Hangzhou Institute of Innovative Medicine, College of Pharmaceutical Sciences, Zhejiang University, Hangzhou 310058, China.

[#] These authors contributed equally to this work.

*** Contact Information of Corresponding Authors:**

Hao Yan, Peihua Luo

Address: 866 Yuhangtang Road, Zijingang Campus, Zhejiang University, Hangzhou, Zhejiang, P.R.China

E-mail: yh925@zju.edu.cn (H. Yan), peihualuo@zju.edu.cn (P. Luo)

Tel: +86-571-88206915

Fax: +86-571-88208400

Supporting information includes 13 Supplementary figures (with legends), 3 Supplementary tables, a set of Original Images for Western Blots, and a set of Original Flow Cytometry Images

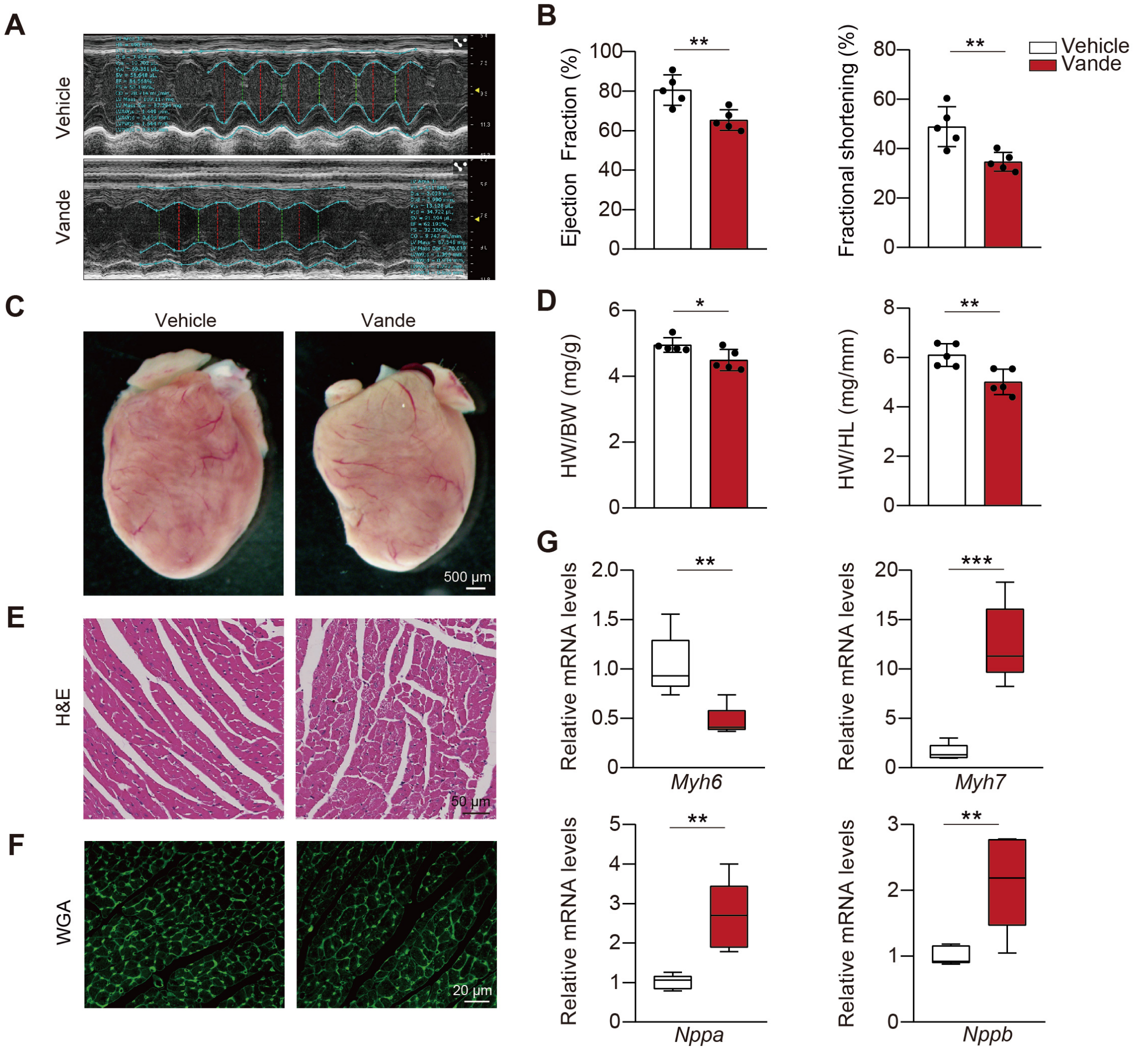

Supplementary figure 1. Vandetanib induced cardiotoxicity. C57BL/6 mice were randomly divided into 2 groups (n = 5 per group). C57BL/6J mice were treated with 0.5% CMC-Na or 100 mg/kg vandetanib for 4 weeks. (**A**) Representative M-mode echocardiogram images were recorded by echocardiography. (**B**) The quantifications of Ejection fraction, Fractional shortening. (**C**) Cardiac tissue diagram. Scale bar, 500 μm. (**D**) Gravimetric analysis of heart weight to body weight ratio (HW/BW) or tibia length ratio (HW/HL) in the indicated groups. (**E**) H&E staining of cardiac tissues. Scale bar, 50 μm. (**F**) WGA staining of cardiac tissues. Scale bar, 20 μm. (**G**) Total RNA was extracted from cardiac tissues and mRNA levels of *Myh6*, *Myh7*, *Nppa* and *Nppb* were

measured by qRT-PCR. (n = 5). Data are represented as the mean ± SD. *p < 0.05, **p < 0.01, ***p < 0.001. Unpaired two-sided Student's *t* test for (**B**), (**D**) and (**G**).

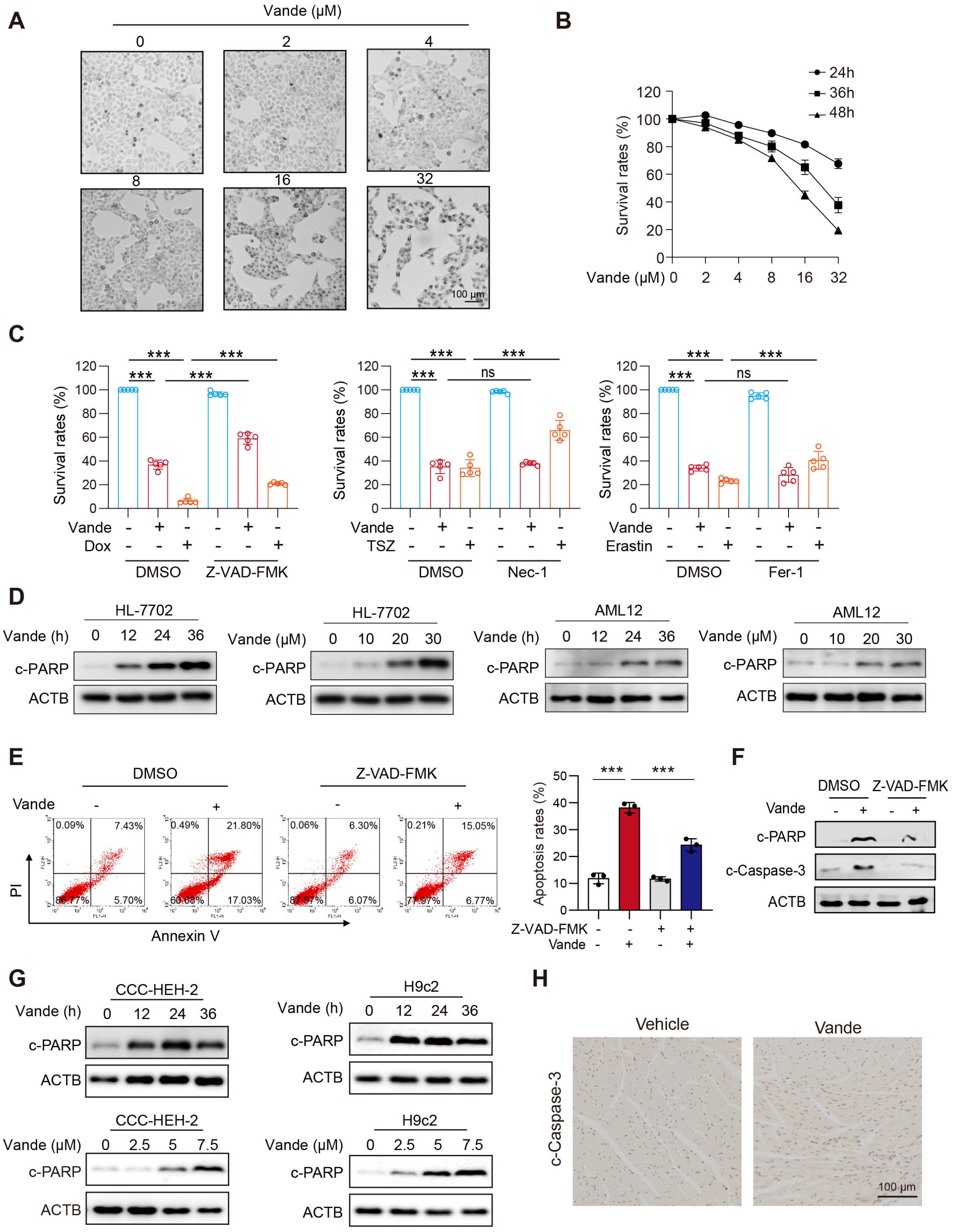

Supplementary figure 2. Vandetanib caused hepatocytes and cardiomyocytes apoptosis. (A)

Representative images of vandetanib-treated HL-7702 cells were photographed by light microscope. Scale bar, 100 μm. (**B**) HL-7702 cells were treated with 0, 2, 4, 8, 16 and 32 μM vandetanib for 24, 36 or 48 hours respectively, and the survival rates were detected via SRB staining. (n = 3). (**C**) HL-7702 cells were treated for 48 h under the following conditions to assess specific cell death pathways: (Left) 20 μM vandetanib or the apoptosis inducer Doxorubicin (DOX, 10 μM), with or without the pan-caspase inhibitor Z-VAD-FMK (20 μM); (Middle) 20 μM vandetanib or the necroptosis inducer TSZ (TNF-α/SMAC-mimetic/Z-VAD-FMK, 1x), with or without the necroptosis inhibitor Necrostatin-1 (Nec-1, 20 μM); (Right) 20 μM vandetanib or the ferroptosis inducer Erastin (20 μM), with or without the ferroptosis inhibitor Ferrostatin-1 (Fer-1, 10 μM). (n = 5). (**D**) HL-7702 cells and AML12 cells were treated with 20 μM vandetanib for 0, 12, 24 or 36 h or 0, 10, 20 or 30 μM vandetanib for 36 h. The expression levels of c-PARP were detected by western blot. (**E**) HL-7702 cells were treated with 20 μM vandetanib and/or 20 μM Z-VAD-FMK for 36 h. Apoptosis rates were analyzed by flow cytometry with Annexin V/PI staining. (n = 3). (**F**) HL-7702 cells were treated with 20 μM vandetanib and/or 20 μM Z-VAD-FMK for 36 h. The expression levels of c-PARP and cleaved Caspase 3 were detected by western blot. (**G**) CCC-HEH-2 cells and H9c2 cells were treated with 0, 2.5, 5 or 7.5 μM vandetanib for 36 h or 5 μM vandetanib for 0, 12, 24, 36 h. The expression levels of c-PARP were measured by western blot. (**H**) The expression levels of cleaved Caspase 3 in cardiac tissues of mice treated with 0.5% CMC-Na or 100 mg/kg vandetanib for 4 weeks were detected by immunohistochemical analysis. Scale bar, 100 μM. Data are represented as the mean ± SD. *p < 0.05, **p < 0.01, ***p < 0.001. One way ANOVA followed by Tukey post hoc test for (**C**) and (**F**).

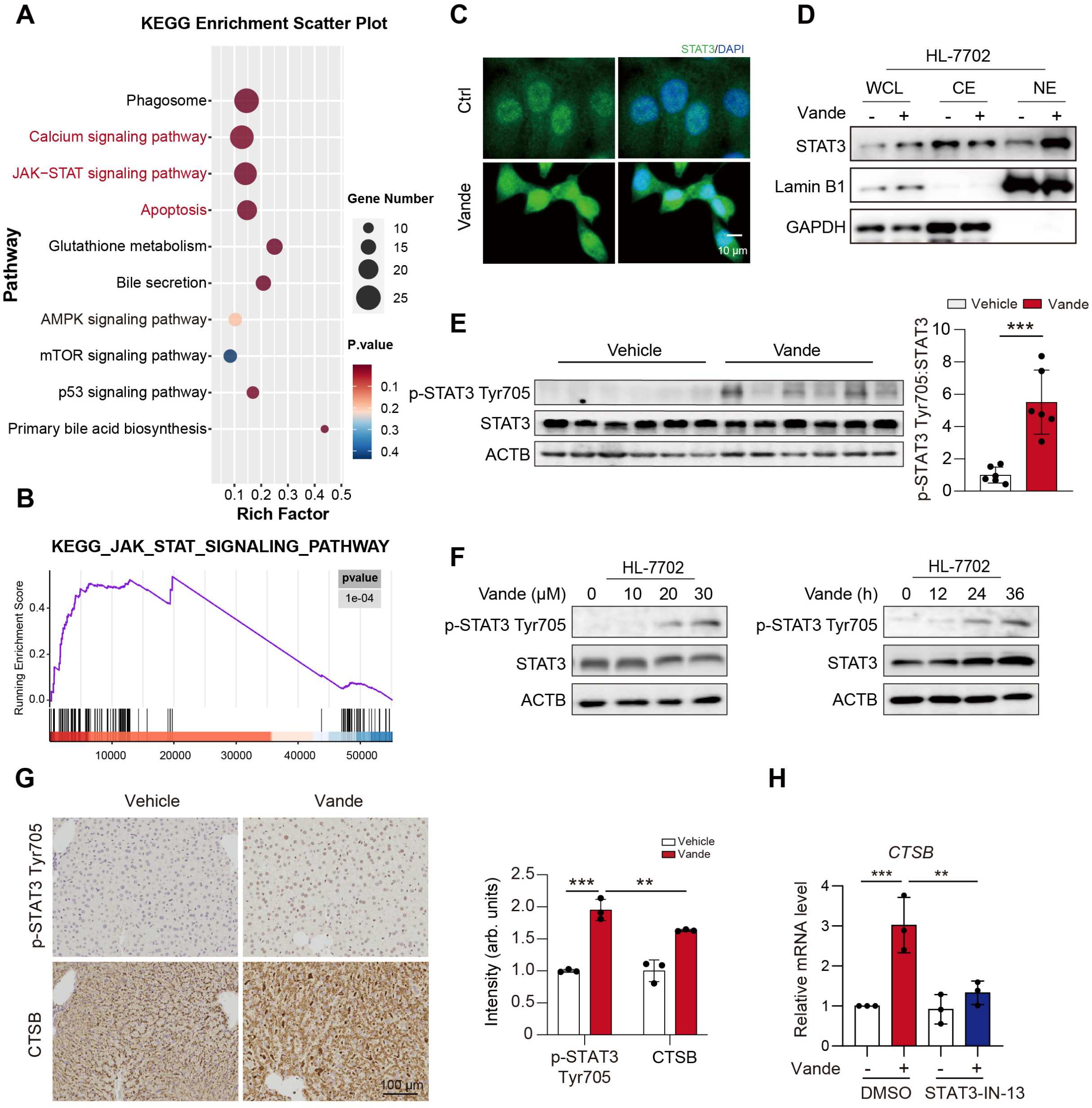

Supplementary figure 3. Vandetanib induced hepatocytes accumulation of CTSB by activating STAT3. (**A**) KEGG enrichment analysis of the changes of signal pathway in liver tissues of mice treated with 0.5% CMC-Na or 100 mg/kg vandetanib for 4 weeks. (**B**) GSEA plots for JAK-STAT signaling pathway significantly enriched after vandetanib's treatment. (**C**) HL-7702 cells were treated with 20 μM vandetanib for 36 h. The location of STAT3 was detected by immunofluorescent assay. Scale bar, 10 μm. (**D**) The expression levels of STAT3 and Lamin B1

in whole cell, cytoplasm and nuclei of HL-7702 cells treated with 20 μM vandetanib were detected by western blot. (**E**) The expression levels of p-STAT3 Tyr705 and STAT3 in liver tissues of mice treated with 0.5% CMC-Na or 100 mg/kg vandetanib for 4 weeks were valued by western blot. (n = 6). (**F**) HL-7702 cells were treated with 0, 10, 20 and 30 μM vandetanib for 36 h or 20 μM vandetanib for 0, 12, 24, 36 h. The expression levels of p-STAT3 Tyr705 and STAT3 in HL-7702 cells were valued by western blot. (**G**) The expression levels of p-STAT3 Tyr705 and CTSB in liver tissues of mice treated with 0.5% CMC-Na or 100 mg/kg vandetanib for 4 weeks were detected by immunohistochemical analysis. Scale bar, 100 μM. (**H**) HL-7702 cells were treated with 20 μM vandetanib and/or 1 μM STAT3-IN-13 for 24 h. The mRNA levels of CTSB were measured by qRT-PCR. (n = 3). Data are represented as the mean ± SD. $^{**}p < 0.01$, $^{***}p < 0.001$. Unpaired two-sided Student's t test for (**E**) and (**G**). One way ANOVA followed by Tukey post hoc test for (**H**). WCL, whole cell lysate; CE, cytoplasmic extract; NE, nuclear extract.

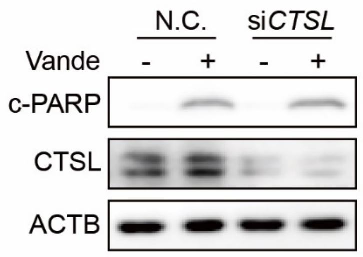

Supplementary figure 4. **Silencing *CTSL* had no effect on vandetanib-induced HL-7702 apoptosis.** HL-7702 cells were transfected with negative control or siRNA targeting *CTSL* and then treated with 20 μM vandetanib for 36 h. The expression levels of c-PARP and CTSL were measured by western blot.

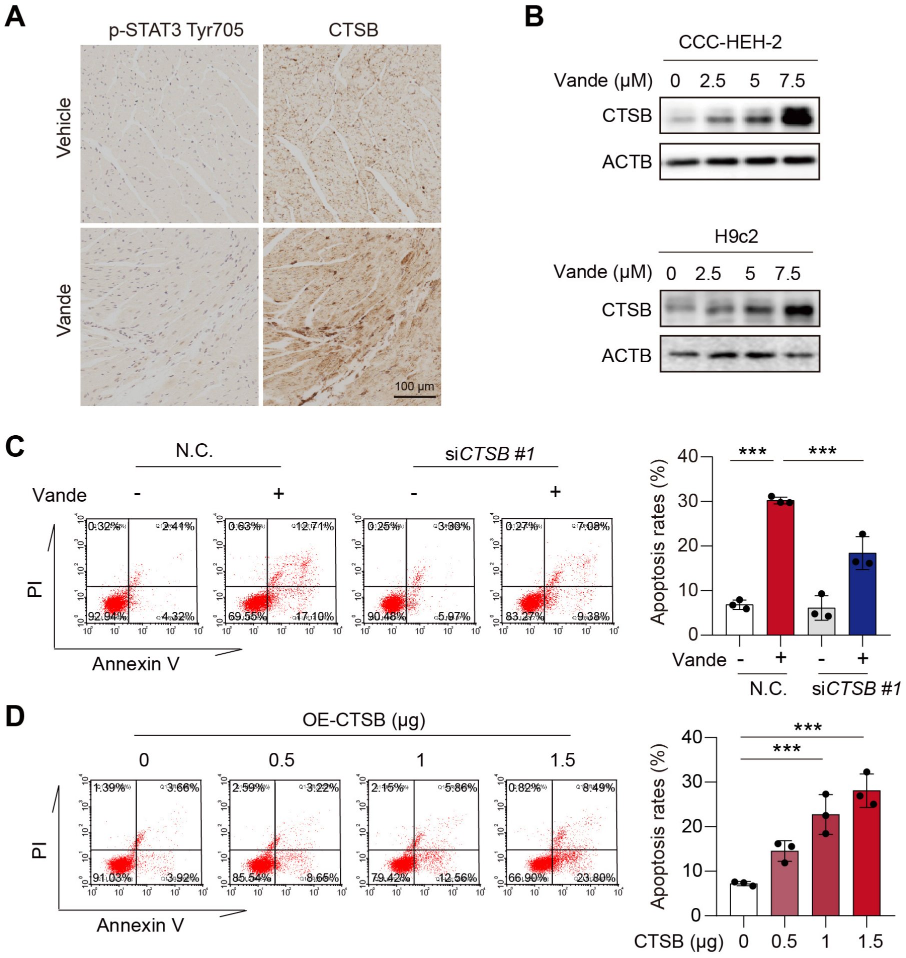

Supplementary figure 5. Vandetanib upregulated CTSB to trigger cardiomyocyte apoptosis. (**A**) The expression levels of p-STAT3 Tyr705 and CTSB in cardiac tissues of mice treated with 0.5% CMC-Na or 100 mg/kg vandetanib for 4 weeks were detected by immunohistochemical analysis. Scale bar, 100 µM. (**B**) CCC-HEH-2 cells and H9c2 cells were treated with 0, 2.5, 5 and 7.5 µM vandetanib for 36 h. The expression levels of CTSB of cells were measured by western blot. (**C**) CCC-HEH-2 cells were transfected with negative control or siRNA targeting CTSB, continued with 20 µM vandetanib for 36 h. Then, apoptosis rates were detected by flow cytometry with Annexin V/PI staining. (n = 3). (**D**) CCC-HEH-2 cells were transfected with vector or 0.5,

1.0 or 1.5 μg pcDNA3.0-CTSB plasmid for 36 h. (n = 3). Data are represented as the mean ± SD. ***$p < 0.001$. One way ANOVA followed by Tukey post hoc test for (**C**) and (**D**).

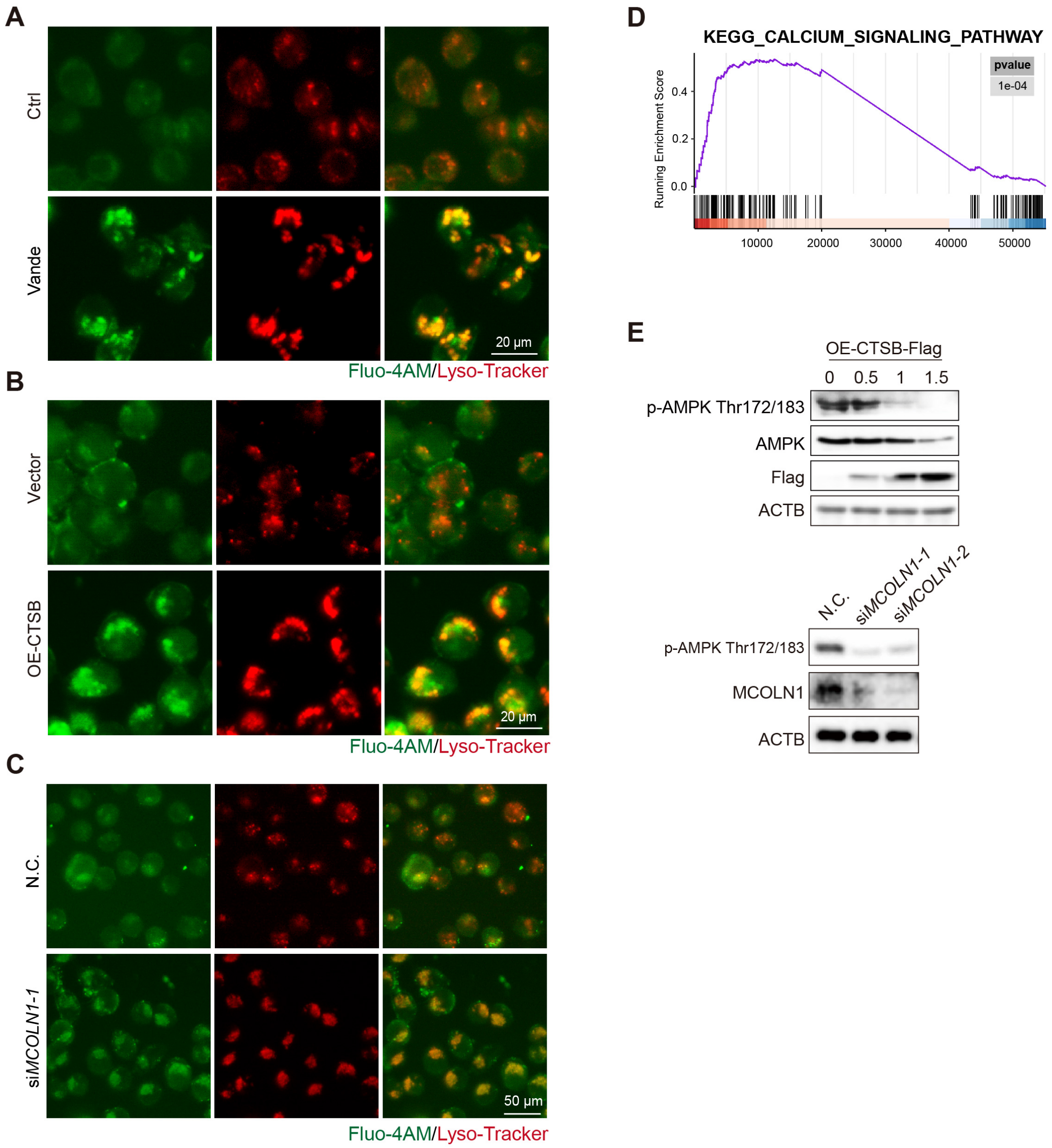

Supplementary figure 6. Vandetanib induces disruption of calcium homeostasis and suppresses AMPK phosphorylation via upregulation of CTSB. (**A**) HL-7702 cells were treated with 20 μM vandetanib for 36 h. The localization of calcium ions was detected by Fluo-4AM staining and the localization of lysosomal was detected by Lyso-Tracker staining. Scale bar, 20 μm. (**B**) HL-7702 cells were transfected with vector or 1 μg pcDNA3.0-CTSB-Flag plasmid for

36 h. The localization of calcium ions was detected by Fluo-4AM staining and the localization of lysosomal was detected by Lyso-Tracker staining. Scale bar, 20 μm. (**C**) HL-7702 cells were transfected with negative control or siRNA targeting MCOLN1 for 36 h. The localization of calcium ions was detected by Fluo-4AM staining and the localization of lysosomal was detected by Lyso-Tracker staining. Scale bar, 50 μm. (**D**) GSEA plots for Calcium signaling pathway significantly enriched after vandetanib's treatment. (**E**) (Upper) HL-7702 cells were transfected with vector or 0.5, 1, 1.5 μg pcDNA3.0-CTSB-Flag plasmid for 24 h. The expression levels of p-AMPK Thr172/183, AMPK and Flag were measured by western blot. (Lower) HL-7702 cells were transfected with negative control or siRNA targeting MCOLN1 for 36 h. The expression levels of p-AMPK Thr172/183, AMPK and Flag were measured by western blot.

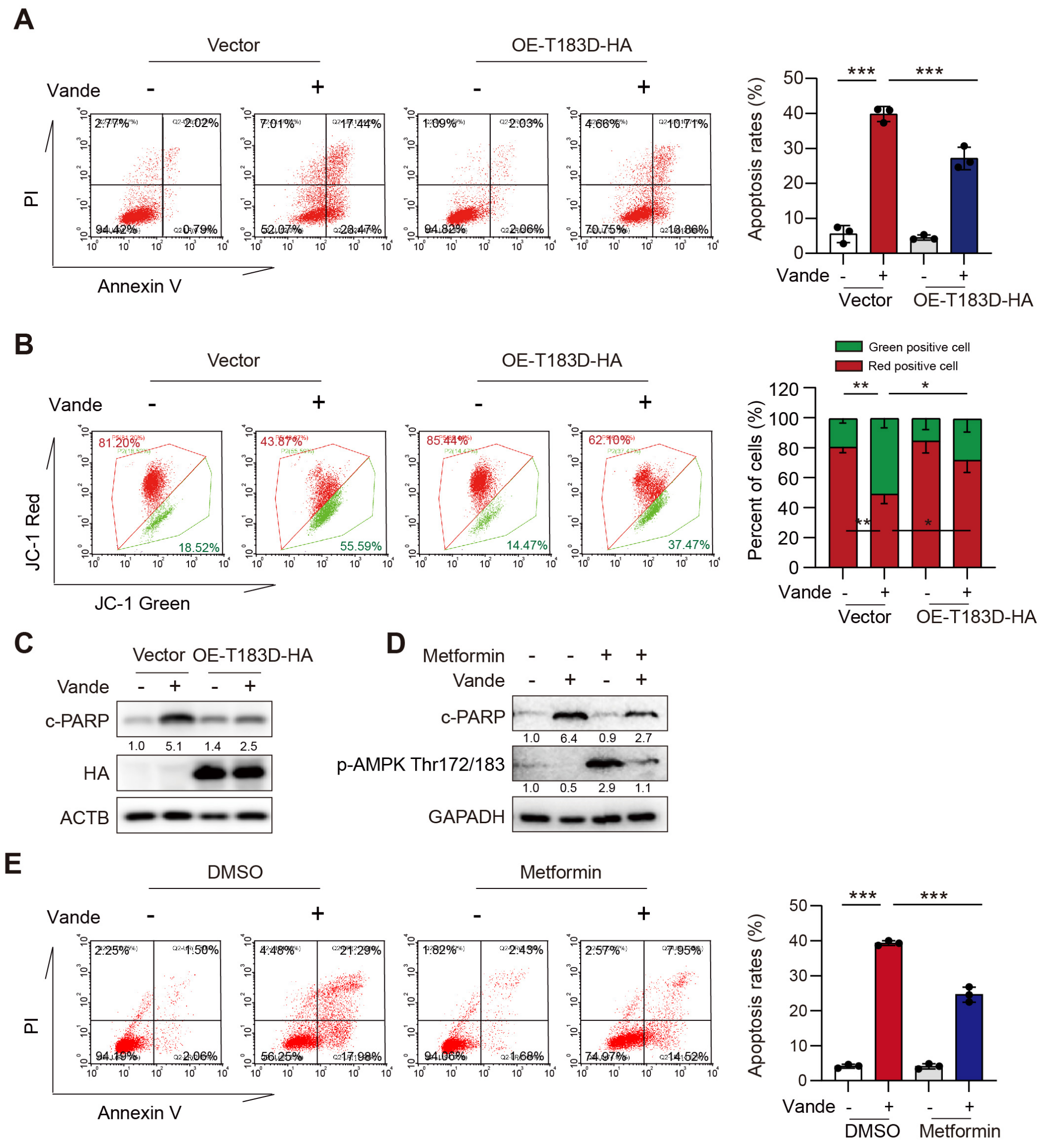

Supplementary figure 7. Sustained AMPK activation mitigates vandetanib-induced hepatotoxicity and mitochondrial damage. (**A-C**) Simulated sustained phosphorylation activation (AMPK-T183D) is performed by mutating AMPK threonine 183 to aspartic acid. Then, HL-7702 cells were treated with 20 μM vandetanib for 36 h. (**A**) The apoptosis rates were detected by flow cytometry with Annexin V-PI staining. (n = 3). (**B**) The mitochondrial membrane potential was detected by flow cytometry with JC-1 staining. (n = 3). (**C**) The expression levels of c-PARP and HA were detected by western blot. (**D-E**) HL-7702 cells were treated with 20 μM vandetanib

and/or 0.5 mM metformin for 36 h. (**D**) The expression levels of c-PARP and p-AMPK T172 were detected by western blot. (**E**) The apoptosis rates were detected by flow cytometry with Annexin V-PI staining. ($n = 3$). Data are represented as the mean ± SD. *$p < 0.05$, **$p < 0.01$, ***$p < 0.001$. One way ANOVA followed by Tukey post hoc test for (**A**), (**B**) and (**E**).

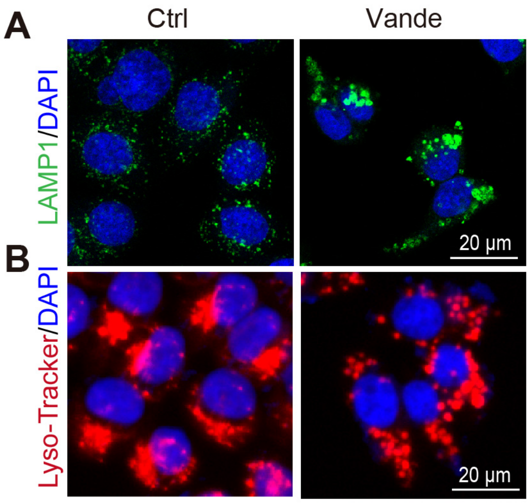

Supplementary figure 8. Vandetanib induces morphological abnormalities in lysosomes. (**A-B**) HL-7702 cells were treated with 20 μM vandetanib for 36 h. (**A**) The expression levels of LAMP1 were measured by immunofluorescence. Scale bar, 20 μm. (**B**) The localization of lysosomal was detected by Lyso-Tracker staining. Scale bar, 20 μm.

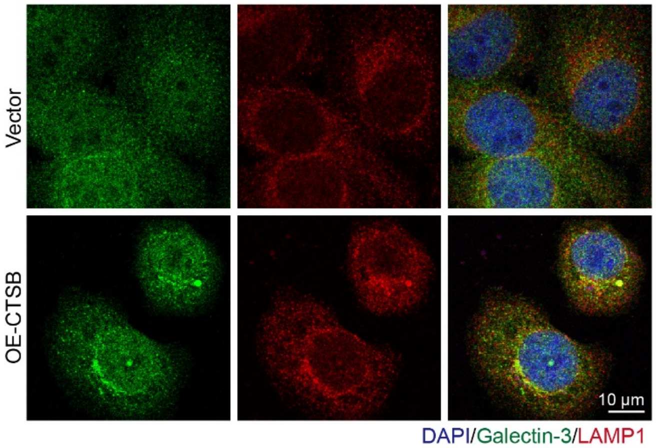

Supplementary figure 9. Overexpression of CTSB damaged lysosomes. HL-7702 cells were transfected with vector or 1 μg pcDNA3.0-CTSB-Flag plasmid for 6 h. The expression levels of Galectin-3 and LAMP1 were measured by immunofluorescence. Scale bar, 10 μm.

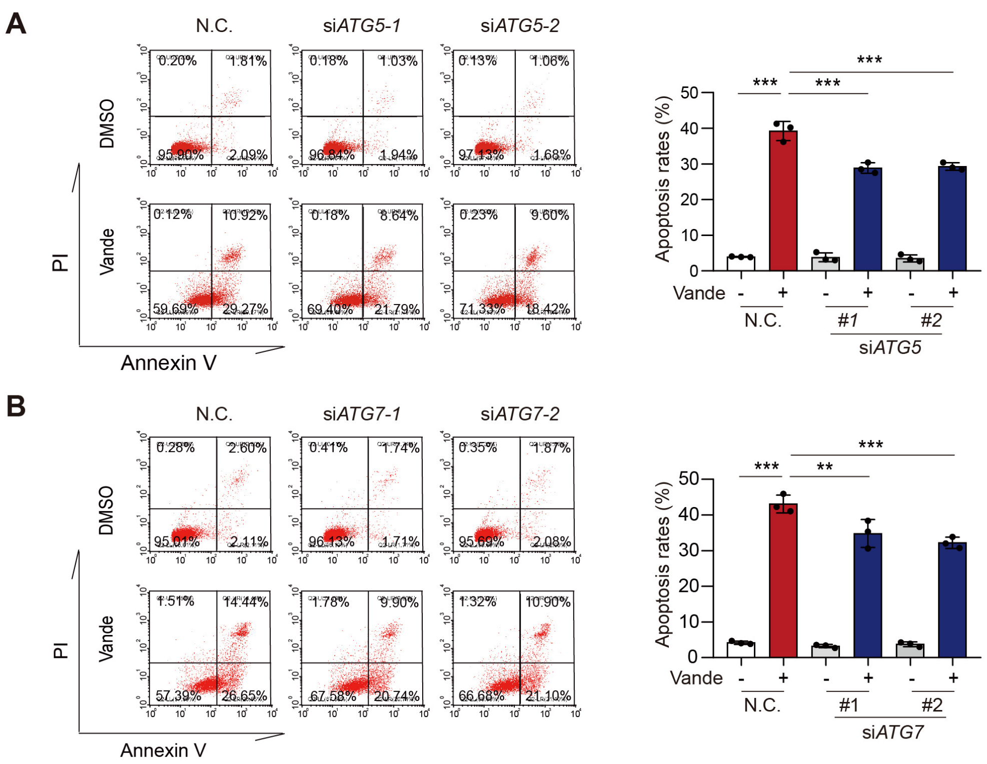

Supplementary figure 10. Silencing *ATG5* and *ATG7* alleviated vandetanib-induced HL-7702 apoptosis. (**A**) HL-7702 cells were transfected with negative control or siRNA targeting *ATG5* and then treated with or without 20 μM vandetanib for 36 h. Then, apoptosis rates were detected by flow cytometry with Annexin V/PI staining. (**B**) HL-7702 cells were transfected with negative control or siRNA targeting *ATG7* and then treated with or without 20 μM vandetanib for 36 h. Then, cells were harvested and stained with Annexin V/PI, apoptosis rates were detected by flow cytometry. Data are represented as the mean ± SD (n = 3). $^{**}p < 0.01$, $^{***}p < 0.001$. One way ANOVA followed by Tukey post hoc test for (A) and (B).

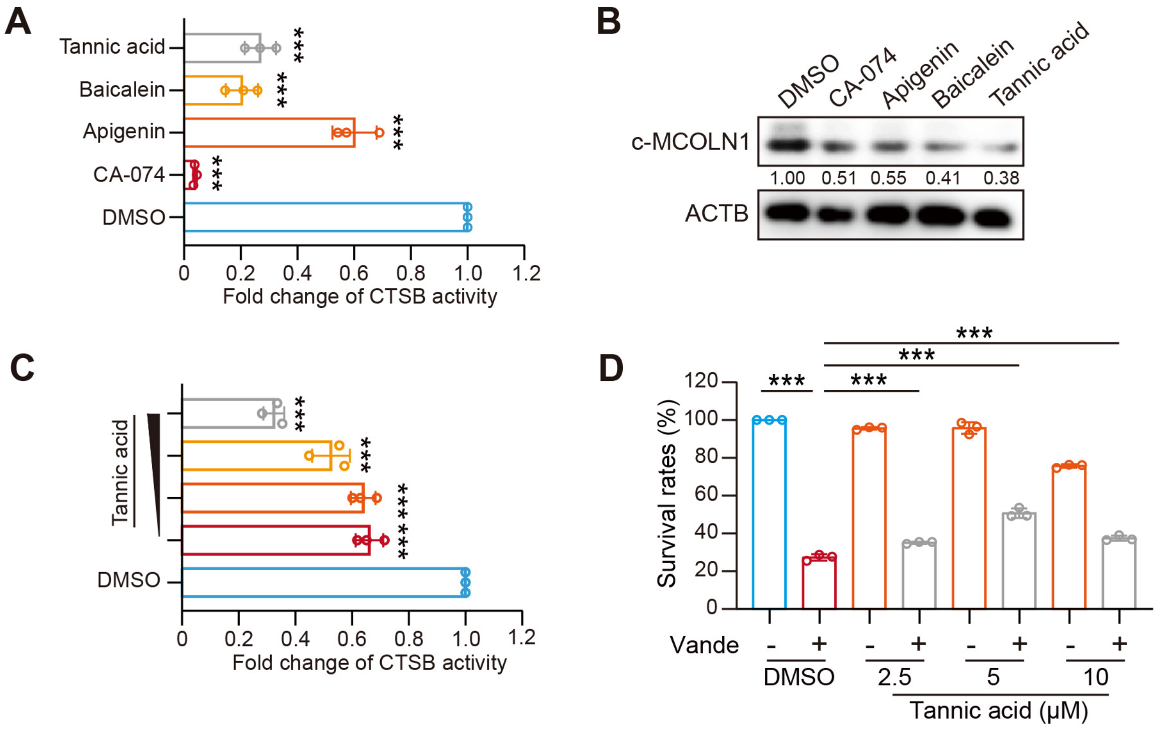

Supplementary figure 11. Tannic acid protects against Vandetanib-induced cell death by inhibiting CTSB activity. (**A**) Protease assays of CTSB in the presence of 250 μM of the indicated compounds. (n = 3). (**B**) HL-7702 cells were treated with 5 μM CA-074, 5 μM Apigenin, 5 μM Baicalein, 5 μM Tannic acid for 36 h. (**C**) The protease activity of CTSB was measured after incubation with increasing concentrations (0, 31.25, 62.5, 125, and 250 μM) of Tannic acid. (**D**) HL-7702 cells were treated with 20 μM vandetanib and/or Tannic acid (0, 2.5, 5, and 10 μM) for 36 h. The survival rates of HL-7702 cells were measured by SRB staining. (n = 3) Data are represented as the mean ± SD. ***p < 0.001. One way ANOVA followed by Tukey post hoc test for (**A**), (C) and (**D**).

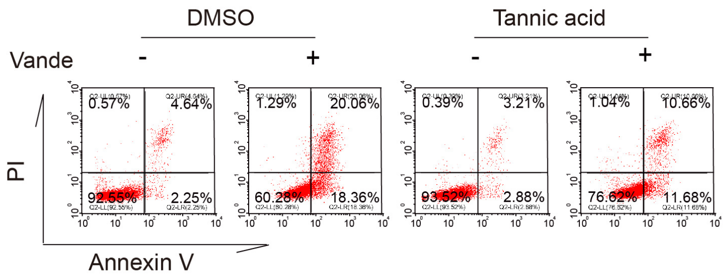

Supplementary figure 12. Tannic acid protects against Vandetanib-induced cell apoptosis. HL-7702 cells were treated with 20 μM vandetanib and/or 5 μM tannic acid for 36 h. Representative flow cytometry plots of Annexin V/PI staining.

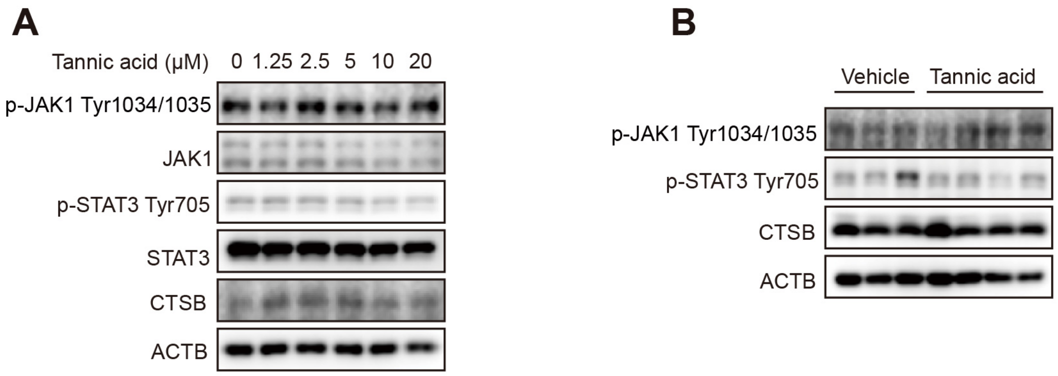

Supplementary figure 13. The administration of Tannic acid did not alter the expression levels of CTSB, p-JAK1, or p-STAT3 *in vivo* and *in vitro*. (**A**) HL-7702 cells were treated with 0, 1.25, 2.5, 5, 10, 20 μM Tannic acid for 36 h. The expression levels of p-JAK1, p-STAT3 and CTSB were measured by western blot. (**B**) C57BL/6J mice were received 30 mg/kg tannic acid by gavage for 4 weeks. The expression levels of p-JAK1, p-STAT3 and CTSB were measured by western blot.

Supplementary table 1. Key resources table

REAGENT or RESOURCE	SOURCE	IDENTIFIER
**Antibodies**		
Anti-STAT3	Cell Signaling Technology	Cat#9139, RRID:AB_331757
Anti-LC3B	Cell Signaling Technology	Cat# 2775, RRID:AB_915950
Anti-LAMP1	Cell Signaling Technology	Cat#15665, RRID:AB_2798750
Anti- Galectin-3	Santa Cruz Biotechnology	Cat#sc-23938, RRID:AB_627658
Anti-cleaved PARP	Huabio	Cat#ET1608-10; RRID:AB_3069785
Anti-cleaved PARP (Asp214)	Cell Signaling Technology	Cat#94885, RRID:AB_2800237
Anti-CTSB	Cell Signaling Technology	Cat#31718, RRID:AB_2687580
Anti-cleaved caspase-3	Cell Signaling Technology	Cat#9664, RRID:AB_2070042
Anti-LC3	MBL	Cat#M152-3; RRID:AB_1279144
Anti-AMPK	Cell Signaling Technology	Cat#2532, RRID:AB_330331
Anti- p-STAT3 (Tyr705)	Cell Signaling Technology	Cat#9145, RRID:AB_2491009
Anti- p-AMPK (Thr172/183)	Cell Signaling Technology	Cat#50081, RRID:AB_2799368
ATG7	Cell Signaling Technology	Cat#8558, RRID:AB_10831194
Anti-HA	Diagbio	Cat#db2603, PRID: AB_3451976
Anti-ACTB	Diagbio	Cat#db14040; PRID:AB_3661715
Anti-GAPDH	Diagbio	Cat#db106, RRID:AB_3271576
Anti-Flag	Yoche Biotechnology	Cat#db7002, RRID:AB_3451977
Anti-rabbit HRP-labeled secondary antibody	Fude Biological Technology	Cat#FDR007, RRID:AB_2934270.
Anti-mouse HRP-labeled secondary antibody	Fude Biological Technology	Cat#FDM007, RRID:AB_2934269.
Alexa Fluor 488-conjugated secondary antibodies	Thermo Fisher Scientific	Cat#A32742, RRID:AB_2762825
Alexa Fluor 594-conjugated secondary antibodies	Thermo Fisher Scientific	Cat#A21206, RRID:AB_2535792

Anti-Lamin B1	Huabio	Cat#ET1606-27, RRID:AB_3069729
Anti-ATG5	Huabio	Cat#ET1611-38, RRID:AB_3070016
Anti-CTSL	Huabio	Cat#HA722063, RRID:AB_3096540
**Cell lines**		
Human hepatocyte cell line (HL-7702)	Jennio Biological Technology	Cat#JNO-048, RRID:CVCL_6926
Human Embryonic Kidney 293 T cells (HEK293T)	Institute of Biochemistry and Cell Biology	N/A; RRID:CVCL_0063
Alpha mouse liver 12 (AML12)	Stem Cell Bank	N/A, RRID:CVCL_0140
Human embryonic cardiac myocyte cell Line (CCC-HEH-2)	National Infrastructure of Cell Line Resource of China	N/A, RRID:CVCL_VU29
Rat embryonic ventricular myoblast cell line (H9c2)	Jennio Biological Technology	Cat#JNO-M0169 RRID:CVCL_0286

Supplementary table 2. Primer sequences of human gene for real-time PCR.

Gene	Forward	Reverse
M-Myh6	AATCCTAATGCAAACAAGGG	CAGAAGGTAGGTCTCTATGTC
M-Myh7	TTGGGAAATTCATCCGAATC	CCAGAAGGTAGGTCTCTATG
M-Nppa	GAGAGAAAGAAACCAGAGTG	GTCTAGCAGGTTCTTGAAATC
M-Nppb	AATTCAAGATGCAGAAGCTG	GAATTTTGAGGTCTCTGCTG
M-Atp6	TCCTAGGCCTTTTACCACATACA	TTTGTGTCGGAAGCCTGTAA
M-Rpl13	CCTGCTGCTCTCAAGGTTGT	GGTACTTCCACCCGACCTC
M-Ctsb	AGACCTGCTTACTTGCTGTG	GGAGGGATGGTGTATGGTAAG
M-Ctsl	AGCAAGAACCTCGACCATG	TTCCATACCCCATTCACTTCC
M-Actin	GTGACGTTGACATCCGTAAAGA	GCCGGACTCATCGTACTCC
H-CTSB	CTTGAAGAGGCTATGTGGTACC	CCCTGGTCTCTGATCTCTTTG
H-CTSL	CAATCAGGAATACAGGGAAGG G	CTGGGCTTACGGTTTTGAAAG
H-ACTIN	CACCATTGGCAATGAGCGGTTC	AGGTCTTTGCGGATGTCCACGT

M: Mouse; H: Human

Supplementary table 3. Oligonucleotide sequences of siRNAs

**Name**		**Sequence (5’→3’)**
Negative control (NC)	sense	UUCUCCGAACGUGUCACGUdTdT
Negative control (NC)	anti-sense	ACGUGACACGUUCGGAGAAdTdT
si*ATG5* #1	sense	UGACUUUUAUGUUUUAAAGCAdTdT
si*ATG5* #1	anti-sense	CUUUAAAACAUAAAAGUCAAGdTdT
si*ATG5* #2	sense	UGAUUACUUGACUUUUAUGUUdTdT
si*ATG5* #2	anti-sense	CAUAAAAGUCAAGUAAUCAAUdTdT
si*ATG7* #1	sense	UUCUUUAGAUCUGCAAAUGUCdTdT
si*ATG7* #1	anti-sense	CAUUUGCAGAUCUAAAGAAGUdTdT
si*ATG7* #2	sense	AAUCUGUUUUAGUGAAAACCUdTdT
si*ATG7* #2	anti-sense	GUUUUCACUAAAACAGAUUGAdTdT
si*CTSB* #1	sense	GGAACUUCUGGACAAGAAAAGdTdT
si*CTSB* #1	anti-sense	UUUCUUGUCCAGAAGUUCCAAdTdT
si*CTSB* #2	sense	GUGACAAUGGCUUCUUUAAAAdTdT
si*CTSB* #2	anti-sense	UUAAAGAAGCCAUUGUCACCCdTdT
si*CTSL* #1	sense	GGUGUUUGUUAGUCAGGAAGAdTdT
si*CTSL* #1	anti-sense	UUCCUGACUAACAAACACCAAdTdT
si*MCOLN1* #1	sense	GCAGCUCCAGUUACAAGAACCdTdT
si*MCOLN1* #1	anti-sense	UUCUUGUAACUGGAGCUGCUUdTdT
si*MCOLN1* #2	sense	GAUCACGUUUGACAACAAAGCdTdT
si*MCOLN1* #2	anti-sense	UUUGUUGUCAAACGUGAUCAGdTdT
